# Aetokthonotoxin, the Causative Agent of Vacuolar Myelinopathy, Uncouples Oxidative Phosphorylation due to Protonophore Activity

**DOI:** 10.1021/acs.chemrestox.5c00147

**Published:** 2025-07-25

**Authors:** Valerie I. C. Rebhahn, Giorgia Del Favero, Robert Rennert, Timo H. J. Niedermeyer

**Affiliations:** ° Institute of Pharmacy, Pharmaceutical Biology, 9166 Freie Universität Berlin, 14195 Berlin, Germany; † Department of Bioorganic Chemistry, 28403 Leibniz Institute of Plant Biochemistry, 06120 Halle (Saale), Germany; ‡ School of Science, 84498 Constructor University Bremen gGmbH, Bremen 28759, Germany; ⊥ Center for Hybrid Nanostructures, Universität Hamburg, Hamburg 22761, Germany; § Department of Food Chemistry and Toxicology, Core Facility Multimodal Imaging, Faculty of Chemistry, University of Vienna, Vienna 1090, Austria; ¶ Vienna Doctoral School in Chemistry (DoSChem), University of Vienna, Vienna 1090, Austria; # Pion Inc., Forest Row Business Park, Forest Row RH18 5DW, United Kingdom

## Abstract

Aetokthonotoxin (AETX) is an emerging environmental toxin produced by the freshwater cyanobacterium *Aetokthonos hydrillicola*. Accumulating in the food chain, it causes vacuolar myelinopathy, a neurological disease affecting a wide range of wildlife characterized by the development of large intramyelinic vacuoles in the white matter of the brain. So far, the mode of action of AETX is unknown. After discovering that AETX is cytostatic and arrests cancer cell lines in the G_1_ phase, metabolomic profiling of AETX-treated cells as well as an assessment of the physicochemical properties of the compound suggested that AETX is a weakly acidic uncoupler of mitochondrial respiration. We confirmed this hypothesis by *in vitro* assays on mammalian cells, finding that AETX has the expected effects on mitochondrial network morphology, mitochondrial membrane potential, and oxygen consumption rate, resulting in affected ATP generation. We confirmed that AETX is capable of transporting protons across lipid bilayers. In summary, we demonstrate that AETX is a protonophore that uncouples oxidative phosphorylation in mitochondria, which is the primary event of AETX intoxication.

## Graphical abstract

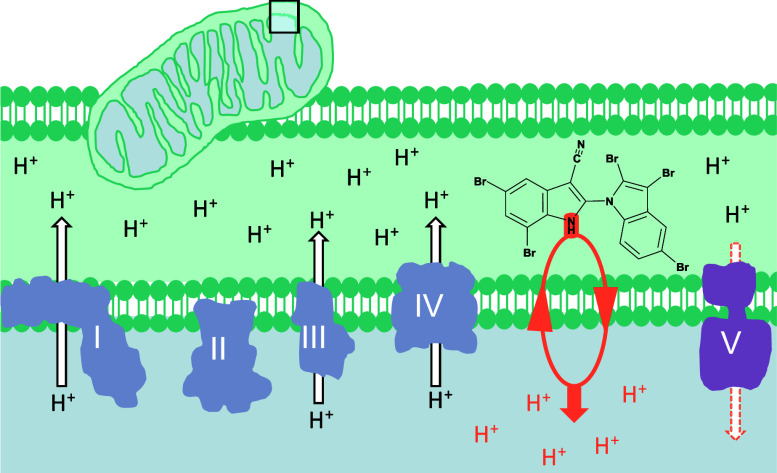

## Introduction

First diagnosed after a mass mortality event of bald eagles (*Haliaeetus leucocephalus*) at DeGray Lake, Arkansas in 1994,^1^ vacuolar myelinopathy (VM) has spread throughout the southeastern United States. Today, it is known that VM affects wildlife of various taxa, apart from birds. These comprise fish, amphibians, and reptiles.^2^ VM is an eventually lethal disease characterized by widespread vacuolization in the white matter of the brain and spinal cord of affected animals with myelinated axons.^2,3^

In 2021, the causative agent of VM was discovered: aetokthonotoxin (AETX, Figure 1). AETX is a naturally occurring toxin synthesized by the freshwater cyanobacterium *Aetokthonos hydrillicola*.^2^ AETX is an unusual pentabrominated biindole alkaloid with a 1,2′-biindole linkage and an indole-3-carbonitrile group, outstanding features for a natural product.^4^ *A. hydrillicola* colonizes the leaves of the invasive neophytic water plant *Hydrilla verticillata*, and wildlife consuming the leaves of the infested plant ingest the AETX-producing cyanobacteria as well.^2^ From there, AETX is passed through the food chain.^2,5,6^ Also, animals without myelin, like mollusks, crustaceans, and nematodes, have been found to be affected by AETX.^2^ As intoxicated animals are debilitated and more likely to be preyed on, AETX poses a severe threat to already endangered predators such as the Florida snail kite (*Rostrhamus sociabilis*).^6^ The mode of action (MoA) of AETX, however, is still unknown. Several structurally different compounds have been linked to the formation of similar edemas in the white matter of the brain.^7^ Despite their structural diversity, most of these compounds seem to interfere with energy metabolism, i.e., oxidative phosphorylation.^7^

**Figure 1:**
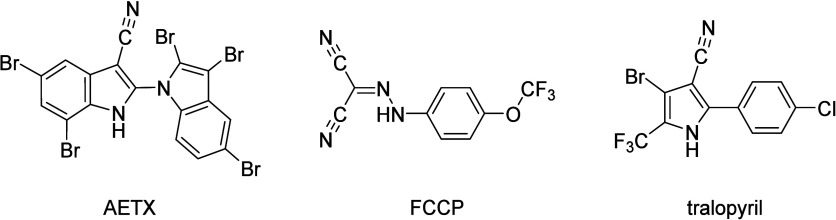
Structures of aetokthonotoxin (AETX), 2-[[4-(trifluoromethoxy)­phenyl]­hydrazinylidene]­propanedinitrile (FCCP), and tralopyril.

A multitude of methods have been developed in the past to elucidate the MoA of compounds, comprising, e.g., chemical proteomics techniques, affinity chromatography-based methods, phage display techniques, or yeast three-hybrid systems.^8,9^ However, many of those methods have the drawback that they require derivatization of the compound of interest to enable, e.g., tagging with biotin or fluorophores.^10^ These modifications often affect the original interaction of the respective compound with its target.^11^ One technique that uses underivatized, genuine compounds is the recently described combination of metabolomics and machine learning for MoA prediction by pattern recognition. This mixed *in vitro*/*in silico* approach is based on the relative quantification of metabolites involved in central carbon and cellular energy metabolism, although downstream effects can also be detected in case compounds affect nonmetabolic targets.^12^

Using this method, we found that the metabolomic changes of AETX-treated cells clustered closely with those of cells treated with uncouplers of oxidative phosphorylation. In addition to a detailed characterization of the impact of AETX on the cellular energy balance and *in vitro* oxygen consumption rate, we show that AETX can act as a protonophore on artificial lipid bilayer membranes. Structure–activity relationships (SAR) for AETX are discussed. Taken together, we confirm uncoupling of the oxidative phosphorylation as the MoA of AETX.

## Experimental Procedures

### Methods—Biology

#### Microbiology

*Escherichia coli* DH5α (DSM 6897) and *Bacillus subtilis* WT168 (DSM 23778) were cultivated on LB-agar plates (Sigma-Aldrich, USA) at 37 °C.

#### Minimal Inhibitory Concentration

The minimal inhibitory concentration (MIC) of AETX was determined using the broth microdilution method according to the guidelines of the Clinical and Laboratory Standards Institute (CLSI).^13^ In short, AETX was dissolved in DMSO and diluted in BD-BBL Mueller–Hinton broth (cation-adjusted, BD, USA) to various concentrations of 0.01–30 μM in a clear 96-well plate (Greiner Bio-One, Germany). The inoculum was produced by picking colonies, suspending them to an optical density (OD_600_) of 0.08–0.10 and diluting 20-fold to inoculate a total of 5 × 10^4^ CFU/well. After an incubation of 20 h with AETX at 37 °C, the bacterial growth was determined by measuring the OD at 620 nm using a Tecan Infinite 200 Pro M Plex plate reader (Tecan Group, Switzerland). An OD_600_ of 0.02 higher than that of the uninoculated control was defined as growth. MICs were determined as the median of biological triplicates with five technical replicates each.

#### Minimal Bactericidal Concentration

The minimal bactericidal concentration (MBC) was investigated according to the CLSI guideline by incubating the 96-well plates described above to a total of 24 h at 37 °C.^14^ Then, 10 μL subcultures, which did not show any visible growth, were streaked onto LB-agar plates. After another 24 h of incubation, the colonies were counted. The determination was performed in biological triplicate with two technical replicates each.

#### Morphological Changes of *B. subtilis*

To visualize the effect of AETX on *B. subtilis* cells, an Olympus CX31 microscope with a PlanC N 40×/0.65 Ph2 objective (Olympus, Japan) was used in dark-field mode after 24 h of incubation for image acquisition. Pictures were taken with a MikrOcular Full HD ocular camera and analyzed using the MikroCamLabII software (both Bresser, Germany).

#### ATP Level of *B. subtilis*

*B. subtilis* was treated for 24 h with AETX in a white, opaque, 96-well plate. The BacTiter-Glo Kit from Promega (Promega, USA) was used according to the manufacturer’s instructions to assess ATP levels. In brief, after the incubation period, the BacTiter-Glo reagent was added in equivalent volumes to the wells. The plate was orbitally shaken for 30 s, and luminescence was recorded after 5 min with a Tecan Infinite 200 Pro M Plex plate reader (Tecan Group, Switzerland).

#### Cell Biology – Cell Lines, Reagents, and Cultivation

Human colorectal cancer HCT116 cells (ACC 581, DMZ-German collection of Microorganisms and Cell Cultures, Germany), human cervix carcinoma HeLa cells (provided by Prof. Junker, Martin Luther University, Halle-Wittenberg, Germany), human foreskin fibroblasts CCD1092Sk cells (immortalized cell line, provided by Prof. Gekle, Julius-Berstein-Institut, Halle, Germany), and human prostate cancer PC-3 cells (purchased from ATCC, USA) were maintained at 37 °C in a humidified atmosphere with 5% CO_2_. Cells were passaged after reaching 80–90% confluence. HeLa and CCD1092Sk cells were cultivated in Dulbecco’s modified eagle medium (DMEM) with low glucose (Carl Roth, Germany) completed with 10% (v/v) fetal bovine serum (FBS, Sigma-Aldrich, USA) and 2 mM glutamine (Carl Roth, Germany), while HCT116 cells were maintained in McCoy’s 5A medium (Carl Roth, Germany) supplied with 10% (v/v) FBS, penicillin (100 IU/mL), and streptomycin (100 mg/L) (Carl Roth, Germany). PC-3 cells were cultured in RPMI 1640 medium, supplemented with 10% (v/v) FBS, 1% (v/v) l-glutamine, penicillin (100 IU/mL), and streptomycin (100 mg/L) (media and supplements purchased from Capricorn Scientific, Germany) and used for a maximum of six passages per batch. AETX and derivatives (purity >95%, Figure S29) were dissolved in DMSO (AppliChem, Germany) and diluted in the respective media used in the individual experiments. For each assay, at least three independent biological replicates were performed (unless stated otherwise). The respective solvent control was always carried out during the course of each experiment.

#### Sulforhodamine B Assay

The sulforhodamine B (SRB) colorimetric assay was conducted as previously described.^15^ Briefly, HeLa cells, fibroblasts, or HCT116 cells were seeded on a clear, cell culture treated 96-well plate with a flat bottom. The next day, the cells were treated with 0.1, 1, and 10 μM of AETX, m-AETX, or dn-AETX, respectively, and incubated for 24 h; fibroblasts were incubated for 48 h, due to their longer doubling time. After the respective incubation time, cells were directly fixed with cold 10% (w/v) trichloroacetic acid for 1 h. Then, cells were carefully rinsed four times with slow-running tap water and blow dried. When cells were completely dry, 0.057% (w/v) SRB solution (Sigma-Aldrich, USA) in 1% (v/v) acetic acid was added to the wells. After 30 min of incubation time at room temperature, the cells were quickly washed four times with 1% (v/v) acetic acid and blow dried. Finally, 10 mM Tris base solution (pH 10.5) was added to the completely dry wells; the plate was placed in a Tecan Infinite M Plex plate reader (Tecan group, Switzerland) and orbitally shaken for 300 s, and absorbance was recorded at 510 nm. In addition, AETX was submitted to the National Cancer Institute Developmental Therapeutics Program. There, AETX was tested on 59 cancer cell lines under use of the SRB assay in a concentration range of 5 nM to 50 μM. To evaluate the toxicity of AETX in dependence on substrate availability, we cultivated HCT116 cells in two different media: DMEM-glucose (DMEM high glucose (25 mM) + 10% FBS + penicillin (100 IU/mL) + streptomycin (100 mg/L) + 2 mM glutamine + 25 mM HEPES) and DMEM-pyruvate (DMEM no glucose + 5 mM pyruvate + 10% FBS + penicillin (100 IU/mL) + streptomycin (100 mg/L) + 2 mM glutamine + 25 mM HEPES) for 24 h before treatment with AETX. AETX was diluted in the respective DMEM-glucose or DMEM-pyruvate medium, and cells were incubated for 24 h with 0.1% DMSO, 0.1, 1, 5, or 10 μM AETX. Then, we conducted the SRB assay as described above.

#### Morphological Changes of HeLa Cells

Phase-contrast microscopy was used to observe the morphological changes of AETX-treated HeLa cells. HeLa cells were seeded in transparent 96-well plates, allowed to settle for 24 h, and then treated for further 24 h with 0.3% DMSO, 0.1, 1, 5, 10, and 30 μM AETX at 37 °C and 5% CO_2_ in a humidified atmosphere. At the end of the incubation time, phase-contrast images were acquired at room temperature and ambient air with an inverse microscope (Axio Observer, Zeiss, Germany), equipped with 10×, 20×, and 63× objective lenses, an Axiocam 712 color digital camera, and ZEN 3.2 software (blue edition, Zeiss, Germany).

#### Confluency Determination

To obtain a first impression of the cytostatic activity of AETX, HCT116 cells were seeded in transparent 96-well plates. After 24 h, confluence was assessed per well using bright-field microscopy to calculate the confluency of the obtained images (Axion Lux microscope and CytoSmart software, both CytoSmart Axion Biosystems, USA). Then, cells were incubated with various concentrations of AETX and imaged again after 24 and 48 h.

#### Cell Cycle Arrest

HCT116 cells were seeded in 12-well plates, allowed to attach overnight, and incubated for 24 h with various concentrations of AETX. Then, cells were detached from the plates, set to 1 × 10^6^ cells per condition, and fixed in ice-cold 70% ethanol. Fixed cells were stored at −20 °C until the day of the flow cytometry experiment. To determine the cell cycle phase, fixed cells were stained for 30 min with propidium iodide solution containing RNase (Thermo Fisher, USA) and analyzed with a CytoFlex flow cytometer (Beckman Coulter, USA) according to the manufacturer’s instructions. To discriminate between cells in the G_0_ and G_1_ phases, fixed cells were stained with *K*_i_-67 FITC-conjugated antibody (#130-117-691, Miltenyi Biotech, Bergisch Gladbach, Germany) for 20 min, washed in PBS, and then analyzed. The software CytExpert v.2.6 (Beckman Coulter, USA) and the free web-based software floreada.io (https://floreada.io) utilizing the Watson pragmatic model were used for gating and cell cycle phase analysis. 2 × 10^4^ cells were analyzed per technical replicate. Three technical replicates per biological replicate were examined. Cells were gated in the following way: First, cell debris was excluded, and cell singlets were selected. Then, a gate was set to separate autofluorescence using unstained cells and the appropriate channel (PE for propidium iodide and FITC for FITC-conjugated *K*_i_-67 antibody).

#### Metabolomics—MTT Assay for Treatment Concentration Determination

Cells were seeded in 96-well plates at 6000 cells per well in 100 μL of medium and allowed to adhere overnight. The cells were then treated with AETX at eight concentrations in 0.5% DMSO. Controls included 0.5% DMSO (negative) and 100 μM digitonin (positive, set as 0% viability; Riedel De Haën, Germany). Each experiment comprised two biological replicates and four technical replicates. After 48 h, the cells were washed with PBS and incubated with MTT solution (0.5 mg/mL; Sigma-Aldrich, Germany) for 1 h under standard growth conditions. Formazan dye, formed by the portion of viable cells, was subsequently dissolved with DMSO, and the absorbance was measured at 570 nm, with 670 nm as a background reference using a SpectraMax M5 plate reader (Molecular Devices, USA). Cell viability was calculated relative to that of untreated controls. Data analysis and IC_50_ calculations were performed using GraphPad Prism 10 and Microsoft Excel 2013, employing a four-parameter logistic function for mean values. IC_50_ was determined to be 0.74 μM AETX (Figure S9). This concentration was subsequently used for the metabolomic assay.

#### Metabolomics—Sample Preparation

Metabolomics samples were proceeded in accordance with the protocol outlined before.^12^ In brief, cells were seeded in T-25 flasks with six replicates for each condition. After adhering overnight, cells were treated with solvent (0.0074% DMSO), or AETX at IC_50_ concentration determined from a previous viability assay (IC_50_ in the MTT assay: 0.74 μM). After 48 h of incubation under standard growth conditions, the untreated control cells reached 85% confluency. In addition, similar independent experiments were conducted using 2, 4, and 24 h incubation time with AETX. Subsequently, the cells were washed with prewarmed PBS (37 °C). Subsequently, cells were rapidly fixed with a cold acidic ethanol solution (10% v/v HCl, pH 1.4, −80 °C). Tightly sealed flasks were submerged in an ultrasonic water bath with dry ice to maintain a low temperature (around 4 °C) while being subjected to 5 min of sonication to detach the cells. The cell suspension was transferred to prechilled Eppendorf tubes on dry ice. Sample volumes were reduced to 50 μL using a nitrogen stream. Two rounds of centrifugation were performed at 10,600 rcf for 5 min at 1 °C, and the supernatants were transferred after each step to fresh tubes. The final supernatant was placed in LC-MS vials and stored at −80 °C until analysis.

#### Metabolomics—Analysis

The metabolomics experiments were conducted following the protocol recently described.^12^ In brief, hydrophilic metabolites were separated using ion-pairing chromatography on a Nucleoshell RP18 column (2.1 × 150 mm, 2.1 μm particle size; Macherey & Nagel, Germany) with a Waters ACQUITY UPLC System, featuring an ACQUITY Binary Solvent Manager and Sample Manager (injection volume 5 μL; Waters, Germany). The mobile phases were 10 mmol/L tributyl amine (pH 6.2, adjusted with glacial acetic acid) for eluent A and acetonitrile for eluent B. The elution profile started with 2% (v/v) of eluent B in A held isocratically for 2 min, followed by a linear gradient increasing from 2 to 36% (v/v) B in A over 16 min. It then continued from 18 to 21 min up to 95% (v/v) B in A, held isocratically from 21 to 22.5 min, and decreased back to 2% (v/v) B in A from 22.51 to 26 min. The flow rate was 400 μL/min, and the column temperature was maintained at 40 °C. Mass spectrometric analysis of the central carbon and cellular energy metabolism metabolites was conducted using targeted MS/MS with multiple reaction monitoring (MRM) on a QTRAP 6500 System (AB Sciex, Germany), operating in negative ionization mode and managed via Analyst 1.7.1 software (AB Sciex, Germany). Key settings included ion spray voltage at −4500 V, nebulizing gas at 60 psi, source temperature at 450 °C, drying gas at 70 psi, and curtain gas at 35 psi. Data processing involved peak integration using MultiQuant software version 3.0.3 (Sciex, USA). To correct for variations of the cell numbers between treatments and controls, the metabolite peak areas were normalized to the total peak area per sample. Each normalized metabolite signal was then divided by the mean of the normalized control signals within the same experiment. The resulting data were log_2_-transformed. Statistical analysis and data visualization were performed using MetaboAnalyst 6.0 software, employing log_2_-normalized data and range scaling for consistency.

#### Immunofluorescence Microscopy

HCT116 cells were seeded on Ibitreat slides for cell imaging (Ibidi, Germany). Cells were treated for 24 h with 0.1, 1, and 5 μM AETX, washed with PBS, and fixed with a 3.5% (w/v) formaldehyde solution for 15 min in the dark. Afterward, cells were washed with PBS once and then with a PBS–glycine solution (0.375 g of glycine in 50 mL of PBS-A), followed by two more PBS washing steps. Subsequently, cells were permeabilized with 0.2% Triton-X in PBS, blocked with 2% donkey serum for 1 h, and incubated with primary anti-TOM20 antibody (diluted 1:500, # sc-17764, Santa Cruz Biotechnology, Dallas, Texas, USA) for 2 h. Then, cells were washed three times with 0.05% Triton-X in PBS and twice with PBS. Cells were incubated with the secondary antibody AlexaFluor 647 donkey antimouse IgG (no. A31571 Invitrogen, Carlsbad, California, USA), diluted 1:1000, overnight. The previous washing steps were repeated, and cells were submerged in two drops of mounting medium containing DAPI (Carl Roth, Germany). Images were acquired with a confocal LSM Zeiss 800 system using a Plan Apochromat 63×/1.4 Oil DICII objective. Image analysis results from the quantification of *n* > 100 regions of interest of the mitochondrial network with ImageJ v. 1.53t. Every experimental condition was measured in quadruplicates.

#### Oxygen Consumption, Proton Efflux, and ATP Production Rate

A Seahorse XFe96 Analyzer (Agilent, USA) was used to determine the cellular oxygen consumption rate (OCR) and proton efflux rate (PER). 2 × 10^4^ HeLa or CCD1092Sk cells were seeded per well in Seahorse XF cell culture microplates (Agilent, USA) and allowed to attach for 24 h. On the day of the experiment, medium was changed to Seahorse XF DMEM, pH 7.4, supplemented with 10 mM glucose, 2 mM glutamine, and 1 μM pyruvate (all Agilent, USA). Bright-field images of the cells were taken at 37 °C and 0% CO_2_ using a Cytation 1 imaging reader (BioTek, USA) to check for confluence. Meanwhile, a Seahorse XF Cell Mito Stress Kit (Agilent, USA) was prepared according to the manufacturer’s instructions, as were treatment concentrations of AETX. The hydrated sensor cartridge was equipped according to the experimental requirements with various concentrations of AETX or solvent control and the kit components. For HeLa cells, 2 μM oligomycin, 0.5 μM 2-[[4-(trifluoromethoxy)­phenyl]­hydrazinylidene]­propanedinitrile (FCCP), and 0.5 μM rotenone + antimycin A (RAA), and for CCD1092Sk cells, 2 μM oligomycin, 2 μM FCCP, and 0.5 μM RAA were applied. The last compound addition was supplemented with 3 μL/mL of Hoechst 33342. These concentrations and seeding densities were determined as optimal in preliminary experiments. After 1 h of incubation at 37 °C without CO_2_, the plate was placed into the Seahorse XFe96 Analyzer to assess OCR and PER at 37 °C. Each measurement (baseline or after compound addition) consisted of 3 min mixing and 3 min measuring and was repeated three times. After the last measurement, fluorescence images were taken with the imaging reader for cell number calculation. OCR and PER were normalized to the number of cells per well. For the 24 h experiment, cells were incubated for 24 h with three concentrations of AETX. On the following day, the experiment was conducted the same way as described above. The software Wave v 2.6.3.5 and Agilent Technologies Cell Imaging v 1.1.0.17 (both Agilent, USA) were used for data analysis. Coupling efficiency was calculated according to Divakaruni et al. and the Agilent user guide RA.4773611111.^16^ The ATP production rate from glycolysis or oxidative phosphorylation was evaluated using the equations for calculation of ATP production rates recently presented by Desousa et al., using PER instead of extra cellular acidification rate (ECAR).^17^ Figure S14 provides a supporting sketch for the interpretation of the results. The total ATP level was determined in an additional luminescence-based assay using the CellTiter-Glo Kit from Promega (Promega, USA) according to the manufacturer’s instructions. In brief, HeLa and CCD1092Sk cells were seeded into opaque-walled 96-well plates and treated the next day with AETX for 24 h in DMEM-glucose or DMEM-pyruvate, as outlined in the methods part Sulforhodamine B Assay. After the incubation time, the cells were allowed to reach room temperature for 30 min. Then, the CellTiter-Glo reagent was added in equivalent volumes to the wells, and the plate was orbitally shaken for 2 min. After 10 min, luminescence was recorded using a Tecan Infinite 200 Pro M Plex plate reader (Tecan Group, Switzerland).

#### Mitochondrial Membrane Potential

To assess the mitochondrial membrane potential, HeLa and CCD1092Sk cells were seeded in a black 96-well plate with a flat, clear bottom and allowed to attach overnight. Cells were then incubated either for 2 or 24 h with various AETX concentrations. Alternatively, to capture and compare immediate effects, the mitochondrial membrane potential was measured instantly after addition of AETX or its derivatives (m-AETX) and (dn-AETX) to the cells. The JC-10 Mitochondrial Membrane Potential Assay Kit for microplates (no. AB112134, Abcam, Cambridge, UK) was used following the manufacturer’s instructions. Fluorescence of JC-10 was recorded with a TECAN Infinite M Plex plate reader at excitation/emission 490/525 and 540/590 nm.

#### Reactive Oxygen Species

The general reactive oxygen species (ROS) status was assessed in HeLa and CCD1092Sk cells using the fluorescent dye DCF-DA (Sigma-Aldrich, USA). Cells were seeded in black 96-well plates with clear flat bottoms (Corning, USA) and allowed to attach overnight. The following day, cells were incubated for 24 h with various concentrations of AETX to assess the long-term effect of AETX. Then, the cells were washed and incubated for 30 min with 50 μM DCF-DA in phenol red-free DMEM (PAN Biotech, Germany). After the cells were washed twice, fluorescence was measured in phenol red-free DMEM at excitation/emission 480/520 nm in a Tecan Infinite M Plex plate reader. For the short-term kinetic measurement, on the day after seeding, cells were directly incubated for 30 min with 50 μM DCF-DA as stated above. After the washing steps, background fluorescence was measured before AETX in various concentrations and 500 μM H_2_O_2_ (positive control) was added to the cells. Fluorescence was recorded directly and 15, 30, 60, and 120 min after treatment, using the same setting as mentioned above.

#### Statistics

Origin software v. 9.6.0.172 (OriginLab Corporation, USA) was used for statistical analysis. First, normality was assessed with the Lillie-Force test for normality. Then, parametric data was assessed using a two-sided Student’s *t* test, while nonparametric data was assessed using a Mann–Whitney test. Sigmoidal fit was calculated using the same software.

### Methods—Chemistry

#### p*K*_a_ Determination

An automated titrator system with an incorporated UV–vis spectrometer (SiriusT3, Pion Inc., USA) was used to acquire the spectrometric data. The optical system consisted of a photodiode array detector with a deuterium lamp and a fiber optic dip probe, and the titrator module comprised a temperature controller (by a Peltier device with an *in situ* thermocouple), a pH electrode, an overhead stirrer, and motorized dispensers for the automatic delivery of assay titrants and reagents via capillaries. The instrumentation was operated using SiriusT3Control software (V2.0). All experiments were carried out at a controlled temperature 25.0 ± 0.2 °C. The pH range of titration assays was set between pH 2.0 to pH 12.0. Prior to use, 0.5 M KOH base titrant was standardized by the titration of approximately 15 mg of potassium hydrogen phthalate, in triplicate. 0.5 M HCl titrant was subsequently standardized against the base titrant. The assay media for p*K*_a_ determination was kept at a constant ionic strength of 0.15 M KCl and under an argon atmosphere. The pH electrode was calibrated daily using the Avdeef-Bucher four-parameter equation.^18^ HPLC-grade methanol cosolvent was used. The aqueous p*K*_a_ was determined by Yasuda-Shedlovsky extrapolation^19^ from mixtures of water and methanol. Data processing and generation of the reported p*K*_a_ values was carried out using SiriusT3Refine software (V2.0).

#### logP Determination

An automated titrator system (SiriusT3, Pion Inc., USA) was used to acquire the potentiometric data. The aqueous p*K*_a_ value of the sample was premeasured for the calculation of logP from the potentiometric data. Ionic strength adjusted (0.15 M KCl) water and 1-octanol (presaturated with ionic strength adjusted water) were added to 0.6–0.8 mg AETX. The p*K*_a_ in water (premeasured aqueous p*K*_a_) and the apparent p*K*_a_ in the presence of octanol (p_o_K_a_) were compared, and the logP was determined.^20^ Using the experimentally determined p*K*_a_ and logP, a lipophilicity profile (logD vs pH) of the compound was calculated. The potentiometric method for the determination of logP has been thoroughly validated.^21,22^

#### Planar Lipid Bilayer Assay

Planar lipid bilayers were formed as described before.^23,24^ Briefly, two homemade black Delrine half-cuvettes of 2.5 mL were used to sandwich a Teflon septum (20 μm thickness, Goodfellow, UK) having an aperture with a diameter of 100 μm. The surroundings were prepainted with hexadecane dissolved in *n*-hexane at 1–5% (v/v), and the compartments (2.5 mL) were dried for 30–35 min in order to evaporate the solvent. Standard Ag-AgCl reference electrodes with a diaphragm (Metrohm) were used to detect the ionic current. One electrode was grounded, whereas the other was linked to the headstage of an Axopatch 200B amplifier (Axon Instruments from Molecular Devices, USA), used for the conductance measurements in voltage clamp mode. The signals were filtered by an onboard low pass Bessel filter at 1 kHz and with a sampling frequency of 10 kHz. Examination of the current recordings was completed using a Clampfit (Axon Instruments). The current–voltage relation of the individual experiments was determined from single averaged currents at given voltages. The experiment was performed in a temperature-regulated (20 °C) and electrically screened room. The bilayers were made by adding 10 μL of 1,2-diphytanoyl-*sn*-glycero-phosphatidyl-choline (Avanti Polar Lipids, USA) at a concentration of 5 mg/mL in *n*-pentane on top of each half-cuvette (area about 1 cm^2^ each). The solution was supplemented with 100 mM KCl and buffered with 10 mM HEPES (Sigma-Aldrich, USA) at pH 7. We first measured the conductance of the bilayer membrane alone, which was negligible. After ensuring a tight membrane, we added 1 μM AETX or FCCP, both dissolved in DMSO, to the aqueous phase. Due to the geometric constraint having a micrometer-sized lipid patch on one side of the cuvette, equilibrium partitioning of AETX or FCCP is difficult to achieve. Compound addition to the lipid phase caused instability in the membranes. To accelerate equilibrium, we repeatedly broke and reformed the lipid bilayer. At lower concentrations, equilibrium was difficult to achieve, whereas higher concentrations resulted in an unstable lipid membrane. To test proton transport, we exchanged buffered solution on the amplifier side and replaced the solution with water. We titrated in several steps HCl and measured the ion current to perform an I–V plot. Smaller pH gradients showed similar trends but with high fluctuations (data not shown).

#### Purification of AETX and Desnitrile-AETX

Aetokthonotoxin (AETX) and desnitrile-AETX (dn-AETX) were isolated from *A. hydrillicola* extract as described before.^25^ AETX and dn-AETX containing biomass was harvested and lyophilized. The biomass was suspended in 50% MeOH (v/v), homogenized by vortexing, treated with an ultrasonic rod (Bandelin, Germany), and extracted on a shaker for 20 min. After centrifugation, the biomass pellet was subsequently extracted again in the same manner with 50% MeOH (v/v) and twice with 80% MeOH (v/v). All supernatants were combined and dried *in vacuo*. The extract was fractionated using flash chromatography on a C_18_ cartridge (CHROMABOND Flash RS 80 C_18_ec, 15–40 μm, 30.9 × 49 mm, Macherey Nagel, Germany) on a preparative HPLC System (Gilson, USA). A binary gradient from 30 to 60% MeOH (v/v) in water in 6 min, 60–100% MeOH (v/v) in water for a further 18 min, and 100% MeOH for 10 min has been used. Fractions were collected every 2 min and dried in a vacuum centrifuge. The fractions containing AEXT and dn-AETX were redissolved in 2 mL of MeCN 80 (v/v) and subjected to semipreparative HPLC (Dionex UltiMate 3000, Thermo Fisher, USA) using a Luna PFP2 column (250 × 10 mm, 5 μm, 100 Å, Phenomenex, USA). The following chromatographic parameters were used: binary gradient from 64 to 98% (v/v) MeCN in water (0.1% formic acid each) at 5 mL/min for 17 min. For purity control, the following chromatographic parameters were used: Kinetex C18 column (50 × 2.1 mm, 2.6 μm, 100 Å, Phenomenex, USA), binary gradient from 5 to 100% (v/v) MeCN in H_2_O (0.1% formic acid each) at 0.4 mL/min in 18 min, 100% MeCN for 2 min, performed on a 1290 Infinity II (Agilent, USA). Purity has been confirmed to be >99.5%.

#### HPLC-HRMS Data Acquisition

HRMS data was acquired on an Orbitrap Exploris 240 mass spectrometer (Thermo Fisher Scientific, USA) equipped with a heated ESI interface coupled to a Vanquish Flex HPLC system (Thermo Fisher Scientific, USA). The chromatographic parameters are as follows: Kinetex C_18_ column (50 × 2.1 mm, 2.6 μm, 100 Å, Phenomenex, USA), binary gradient from 5 to 100% (v/v) MeCN in water (0.1% formic acid each) at 0.4 mL/min in 16 min, 100% MeCN in 4 min. HRMS data acquisition was conducted in positive and negative ionization modes, ESI spray voltage of 3.5 and −2.5 kV, capillary temperature of 300 °C, sheath gas flow rate of 40 L/min, and auxiliary gas flow rate of 5 L/min. Full-scan accurate mass spectra were acquired from *m*/*z* 133.4 to 2000 with a resolution of 35,000 at *m*/*z* 200.

#### Methylation of AETX

AETX was dissolved in THF. Under stirring at room temperature, dimethyl sulfate (3.0 equiv) and potassium carbonate (5.0 equiv) were added. After it was stirred for 30 min at room temperature, the mixture was concentrated under reduced pressure and purified by HPLC. *R*_f_ (toluene/hexane 2:1) = 0.7.

#### NMR Data Acquisition

NMR spectra were recorded in DMSO-*d*_*6*_ on a Bruker Avance700 (Bruker BioSpin, Germany) operating at 700 MHz (^1^H) or 175 MHz (^13^C) at 300 K. Chemical shifts are in ppm relative to the residual solvent chemical shifts (δ_H_ 2.50, δ_C_ 39.52). NMR data were analyzed with MestReNova (version 14.3.0–30573, Mestrelab Research, Spain) after Auto Phase Correction and Auto Baseline Correction.

#### Quantification of Desnitrile-AETX (dn-AETX) and *N*-Methyl-AETX (m-AETX)

The concentrations of test compound solutions for bioactivity testing were quantified using HPLC coupled with an evaporative light scattering detector (1290 Infinity II, Agilent, USA), as described previously.^26^ Synthetic AETX was used as standard to establish a calibration curve from 23.7 to 237 ng of on-column. 1, 2.5, 5, and 10 μL of a 23.7 ng/μL solution in 90% (v/v) MeCN in H_2_O were injected in triplicate on a Kinetex C18 column (100 × 3 mm, 2.6 μm, 100 Å, Phenomenex) and eluted with a gradient from 10 to 100% (v/v) MeCN in H_2_O (0.1% FA each) over 10 min at 0.65 mL/min.^27^ Settings of the ELSD were as follows: evaporator temperature 45 °C, nebulizer temperature 45 °C, gas flow rate 1.3 standard liter per minute, and N_2_ 3.5 bar. The calibration curve was generated as described by Young and Dolan.^28^ In brief, the response areas were averaged, and log­(ELSD response area) was plotted against log­(amount in nanograms) to generate a linear calibration curve. dn-AETX and m-AETX were dissolved in 1 mL of MeCN 90% (v/v in ddH_2_O), diluted 1:3 in the same solvent, and injected in triplicate under the same conditions.

## Results and Discussion

### AETX Is Bacteriostatic in *Bacillus subtilis* and Cytostatic in Cancer Cell Lines, Arresting Them in the G_1_ Phase

Although the toxicity of AETX has been confirmed *in vivo* in birds (*Gallus gallus*), zebrafish (*Danio rerio*), and *Caenorhabditis elegans*,^2^ its activity on bacteria or mammalian cell lines has not yet been studied in detail. To assess its effect on bacteria, we evaluated the optical density of *B. subtilis* and *Escherichia coli* suspensions after 20 h of incubation with various AETX concentrations. While only a slight reduction of *E. coli* growth was observed at the highest concentration tested (30 μM), the minimal inhibitory concentration (MIC) of AETX for *B. subtilis* was 0.5 μM. However, even after a 24 h incubation time with 30 μM, AETX had no bactericidal but bacteriostatic effect on *B. subtilis*. Dark-field microscopy of *B. subtilis* revealed a change of morphology from rod to spheric shape when treated with AETX at concentrations below the MIC (Figure S1A). In addition, we observed a dose-dependent decrease of ATP in *B. subtilis* after 24 h treatment with AETX (Figure S1B).

Toxicity of AETX for mammalian cells was tested by using a sulforhodamine B (SRB) cytotoxicity assay with the NCI-60 panel of the National Cancer Institute (NCI) Developmental Therapeutics Program (five-dose assay, 5 nM to 50 μM),^29^ including colorectal cancer HCT116 and prostate cancer PC-3 cells, which were later also used by us for more detailed characterization. In addition, cytotoxicity was also determined in cervix carcinoma HeLa cells and in fibroblast CCD1092Sk cells using the SRB assay. In most of the NCI-60 panel cell lines, AETX only showed moderate activity, with half-maximal lethal concentration (LC_50_) values of >50 μM, while the half-maximal growth inhibition (GI_50_) was around 1 μM, and the total growth inhibition (TGI) around 5 μM (Figure S2). We determined a half-maximal effect concentration (EC_50_) of 6.5 μM for HeLa cells (Figure S3), which is in line with the aforementioned TGI determined by the NCI and further agrees with our previously published data.^30^ Based on our results, the tested cancer cell lines seem to be more susceptible to AETX than the noncancerous fibroblast cell line, as we were unable to determine an EC_50_ for the latter (Figure S4).

Interestingly, visual examination of AETX-treated cells during routine bright-field microscopy of the assay plates revealed morphological changes of the cells treated with higher concentrations of AETX, rather than signs of cell death (Figure S5). Along with the results of the SRB assays, the data indicated a cytostatic effect of AETX. To assess this hypothesis, we monitored the confluence of HCT116 cells before and after treatment with AETX. Indeed, we found a steady confluence level of cells treated with higher AETX concentrations starting at 5 μM AETX in contrast to control cells or cells treated with lower AETX concentrations (Figure S6). This finding corresponds well to the TGI of HCT116 cells, as determined by the NCI.

To investigate this finding in more detail, we studied whether the cells were arrested in a certain phase of the cell cycle. Flow cytometric analysis of HCT116 cells treated for 24 h with solvent control, 1 or 5 μM AETX, followed by DNA staining with propidium iodide, revealed an accumulation of the cells in the G_0_/G_1_ phase for both AETX concentrations (Figure S7). To further distinguish if cells accumulate in the G_0_ or G_1_ phase, we stained HCT116 cells with an FITC-conjugated *K*_i_-67 antibody. As only proliferating cells possess this marker (i.e., cells that are not in the G_0_ phase), a decrease in the *K*_i_-67 signal compared to the control would signify an arrest of cells in the G_0_ phase. As we found no significant difference between treated cells and the control (Figure S8), we concluded that the cells were arrested in the G_1_ phase. The cell cycle progression through this phase has been linked to mitochondrial bioenergetics before.^31^

### Metabolomic Profiling and Physicochemical Properties Suggest Uncoupling as MoA of AETX

To generate a first hypothesis about the potential MoA of AETX, its impact on the cells’ central carbon and energy metabolism was studied by metabolomic profiling of PC-3 cells incubated for 2, 4, 24, and 48 h with 0.74 μM AETX (EC_50_ in an MTT cell viability assay, Figure S9). Using a previously published approach, the effects of AETX on the treated cells’ metabolome were compared with the effects of a panel of reference compounds with known MoAs.^12^

When cells were treated for 2 or 4 h, we found that the AETX treatment clustered hierarchically most closely with the outcome of treatments with [(3-chlorophenyl)­hydrazono]­malononitrile (CCCP), 2,4-dinitrophenol (DNP), and emodin, all classified as uncouplers of oxidative phosphorylation (Figure 2 and Figure S10).^12^ After 24 and 48 h of treatment, AETX additionally clustered with hexachlorophene and bithionol (Figure 2 and Figure S10), which, although they are classified as glutamate dehydrogenase (GDH) inhibitors in our model,^12^ are also uncouplers of oxidative phosphorylation.^32,33^ In addition, adenosine monophosphate (AMP), the general marker of a reduced cellular energetic status, had a higher abundance in treated cells than in control cells (Figure S11). In total, these findings underline the interference of AETX with cellular energy homeostasis and suggest the uncoupling of the oxidative phosphorylation as the primary MoA of AETX.

**Figure 2:**
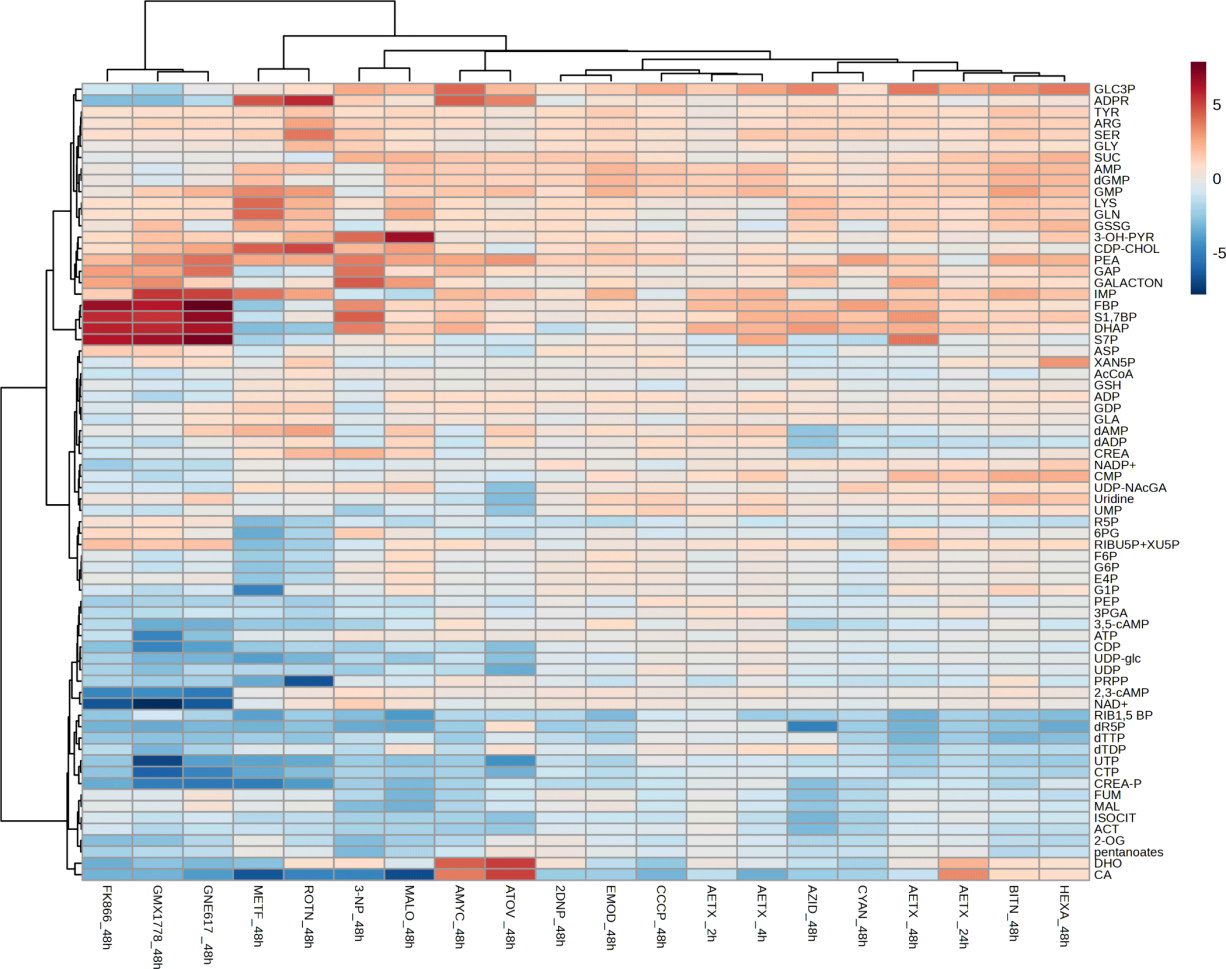
Relative abundance of key intermediates of the central carbon and cellular energy metabolism upon oxidative phosphorylation inhibition at complexes I–IV, treatment with uncouplers, NAMPT inhibition, GDH inhibitors, and AETX treatment. The data represents the average log2-fold changes in peak areas that are relative to the cell number, after 48 h of reference drug treatment, and 2, 4, 24, and 48 h of AETX treatment (*n* = 6), compared to a vehicle control (*n* = 6). Compounds (mode of action): 2DNP—2,4-dinitrophenol (uncoupler), 3-NP—3-nitropropionic acid (CPLX II), AMYC—antimycin A (CPLX III), ATOV—atovaquone (CPLX III), AZID—sodium azide (CPLX IV), BITN—bithionol (GDH), CCCP—carbonyl cyanide chlorophenylhydrazone (uncoupler), CYAN—potassium cyanide (CPLX IV), EMOD—emodin (uncoupler), FK866—FK866 (NAMPT), GMX—GMX1778 (NAMPT), GNE—GNE-617 (NAMPT), HEXA—hexachlorophene (GDH), MALO—malonic acid (CPLX II), METF—metformin (CPLX I), ROTN—rotenone (CPLX I). Abbreviations: CPLX I—complex I, CPLX II—complex II, CPLX III—complex III, CPLX IV—complex IV, GDH—glutamate dehydrogenase, NAMPT—nicotinamide phosphoribosyltransferase, Uncoupler—uncoupling of oxidative phosphorylation. For abbreviations of metabolites, see supplementary metabolomics data.

We subsequently examined the chemical structures and physicochemical properties of known uncouplers. Many uncouplers, including CCCP and DNP, belong to a group called weakly acidic uncouplers. These molecules are, in general, characterized by high lipophilicity in conjunction with a weakly acidic functional group. Due to these properties, weakly acidic uncouplers can transport protons across biological membranes (protonophore activity), with oxidative phosphorylation in mitochondria.^34,35^ Indeed, AETX perfectly matches these general requirements: Its p*K*_a_ value is 6.9 (Figure S12), its logP is 4.7, and its logD at pH 7 is 4.4 (Figure S13). According to Gange et al.,^36^ the optimum p*K*_a_ and logP values for uncoupling are 7.2 and 5.5, respectively, which is close to the values we determined for AETX. We further observed that the structure of tralopyril (Figure 1), the active form of the prodrug chlorfenapyr, is strikingly similar to that of AETX. Both contain an aromatic NH group (indole in AETX, pyrrole in tralopyril) where the mesomeric effect of a nitrile group increases NH acidity, both feature an aromatic system adjacent to the NH-containing heterocycle, and both show halogen substitution that further increases NH acidity due to electron withdrawal from the aromatic system and also increases lipophilicity. Approved as a biocide for restricted uses, chlorfenapyr exerts pan-species toxicity with a high vulnerability of birds.^37^ Trials in rodents showed similar effects and lesions in the brain and spinal cord as observed in animals with VM evoked from AETX.^38^ Likewise, the active metabolite of bromethalin, desmethylbromethalin, is an uncoupler that causes VM-like lesions in cat and bird brains.^39,40^ Taken together, the structural and physicochemical evaluations support the hypothesis of uncoupling as MoA of AETX.

### AETX Shows Uncoupling—Typical Effects on Mitochondria in Mammalian Cells

Mitochondrial bioenergetics is tightly linked to mitochondrial network morphology and *vice versa.* ^41^ To validate the hypothesis and predictions discussed above *in vitro*, we studied how AETX affects the mitochondrial network in HCT116 cells. Immunofluorescence staining of the mitochondrial translocase of the outer membrane (TOM20) revealed a dose-dependent decrease in the intensity of the TOM20 signal (Figure 3A,B). Thus, AETX disrupts the mitochondrial network, visible already at the lowest concentration tested (0.1 μM). This result agrees with previous findings that compounds interfering with oxidative phosphorylation lead to mitochondrial fragmentation in most cell lines tested.^42^

**Figure 3:**
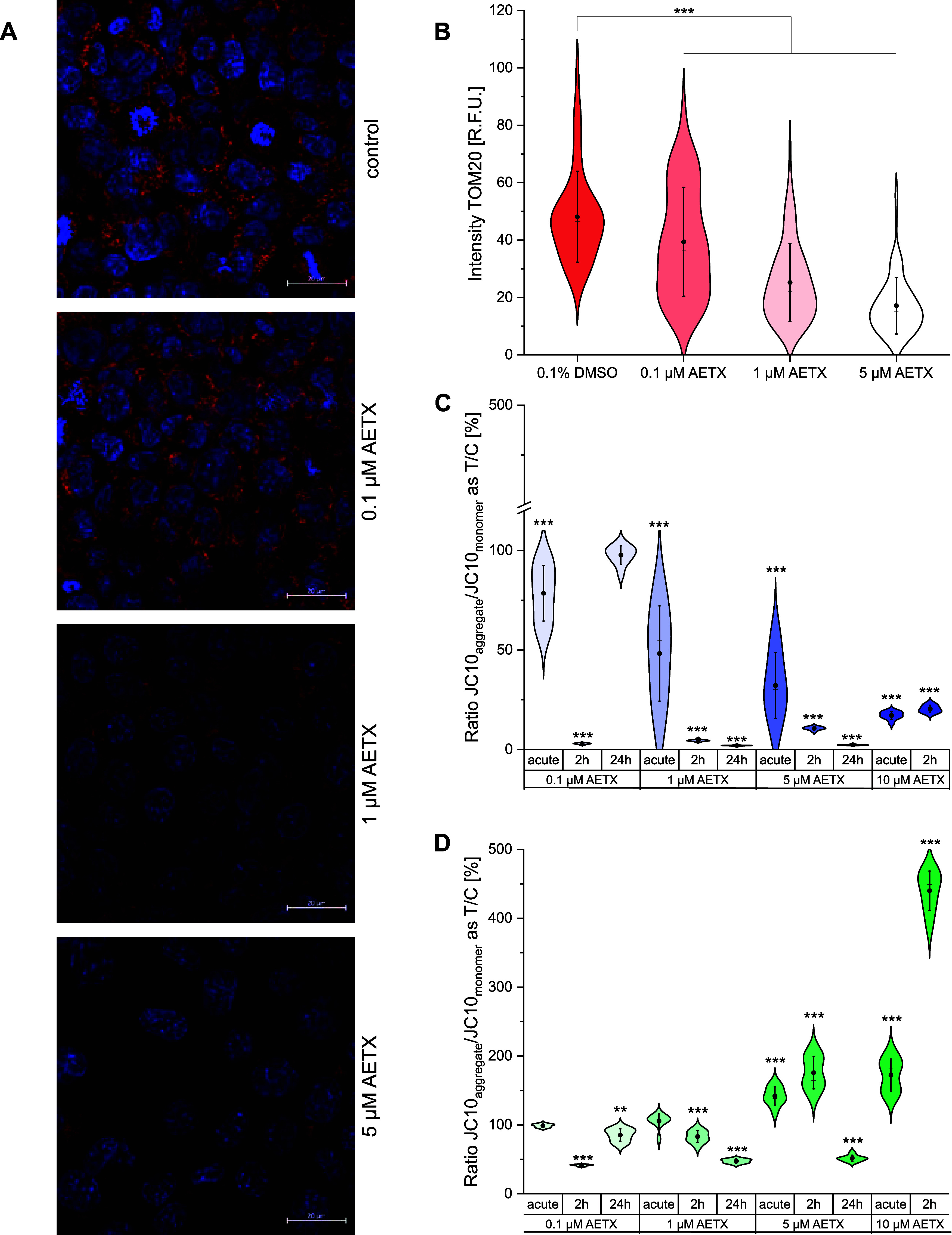
Impact of AETX on the mitochondrial network and membrane potential. (A) Confocal microscopy images of immunofluorescence-stained translocase of the outer mitochondrial membrane 20 protein (TOM20) (red) and DNA (blue) in HCT116 cells. Cells incubated for 24 h with (from top to bottom) 0.1% DMSO (control) or 0.1, 1, or 5 μM AETX. Scale bar 20 μm. (B–D) Violin plots with the mean indicated as a black dot and the median as a line. Standard deviation (1×) indicated as whiskers. Increasing shades of color indicate increasing AETX concentrations. (B) Intensity of the TOM20 signal as relative fluorescence units (R.F.U.) in HCT116 cells. Statistically significant difference to the control indicated with ***p < 0.001, obtained with Student's *t* test. Mitochondrial membrane potential measurement at different time points as the ratio of JC10 aggregate to JC10 monomer in (C) HeLa cells or (D) fibroblasts normalized to control. Statistically significant difference to the control indicated with **p < 0.01, ***p < 0.001, obtained with Mann–Whitney (HeLa cells, 24 h incubation time) or Student's *t* test.

As discussed above, weakly acidic uncouplers, acting as protonophores, enable short-circuiting of protons across the inner mitochondrial membrane. Thereby, the proton motive force (pmf), which is formed by an interplay of the mitochondrial membrane potential (Δψ_m_) and a proton gradient (ΔpH) across the membrane that drives the F_o_F_1_-ATPase, is dissipated. Thus, uncouplers inhibit the biosynthesis of ATP from ADP and inorganic phosphate.^16,34^ Consequently, to restore the pmf, affected cells increase the respiratory chain activity. This comprises an enhanced substrate flux and increased oxygen consumption. Below a certain Δψ_m_ threshold, the F_o_F_1_-ATPase reaction is reversed from ADP phosphorylation to ATPase activity, which further depletes the ATP pool of affected cells.^16^

The oxygen consumption and proton efflux rates (OCR and PER, respectively) of AETX-treated HeLa cells and fibroblasts were determined using a Seahorse XFe96 analyzer (Figure S14). Δψ_m_ was determined using the ratiometric fluorescent dye JC-10 in a plate reader-based assay. Both cell lines have been used in the past to compare effects on mitochondria.^43^ With sufficient glucose supply, like in our experiment, HeLa cells mainly rely on anaerobic glycolysis for ATP synthesis,^44^ while fibroblasts are known to largely rely on oxidative phosphorylation.^45^

First, the OCR and PER were measured directly after the addition of AETX to the cells, followed by the addition of oligomycin, 2-[[4-(trifluoromethoxy)­phenyl]­hydrazinylidene]­propanedinitrile (FCCP), and rotenone/antimycin A (RAA). In both cell lines, the effects of AETX were dose-dependent throughout the experiments. Both cell lines showed an increase of OCR after acute injection of AETX (Figure 4A,B, data on PER see Figures S15–S20). Although the magnitude of the effect was different, subsequent injection of oligomycin led to a decreased OCR and an increased PER in both cell lines, and the effect of FCCP on OCR and PER was diminished dose-dependently after AETX injection. Calculations according to Divakaruni et al.^16^ revealed that both cell lines showed a significantly increased proton leak (Figure S21) and decreased coupling efficiency (Figure S22) compared to the control after treatment with AETX. These findings are indicative of uncoupling activity.^16^ To exclude any other cellular process apart from mitochondrial respiration being responsible for the observed increased oxygen consumption after AETX injection, we first inhibited respiratory chain activity with RAA before adding AETX to the cells (Figure S23). We found no change in OCR, which is typical for uncouplers.^34^

**Figure 4:**
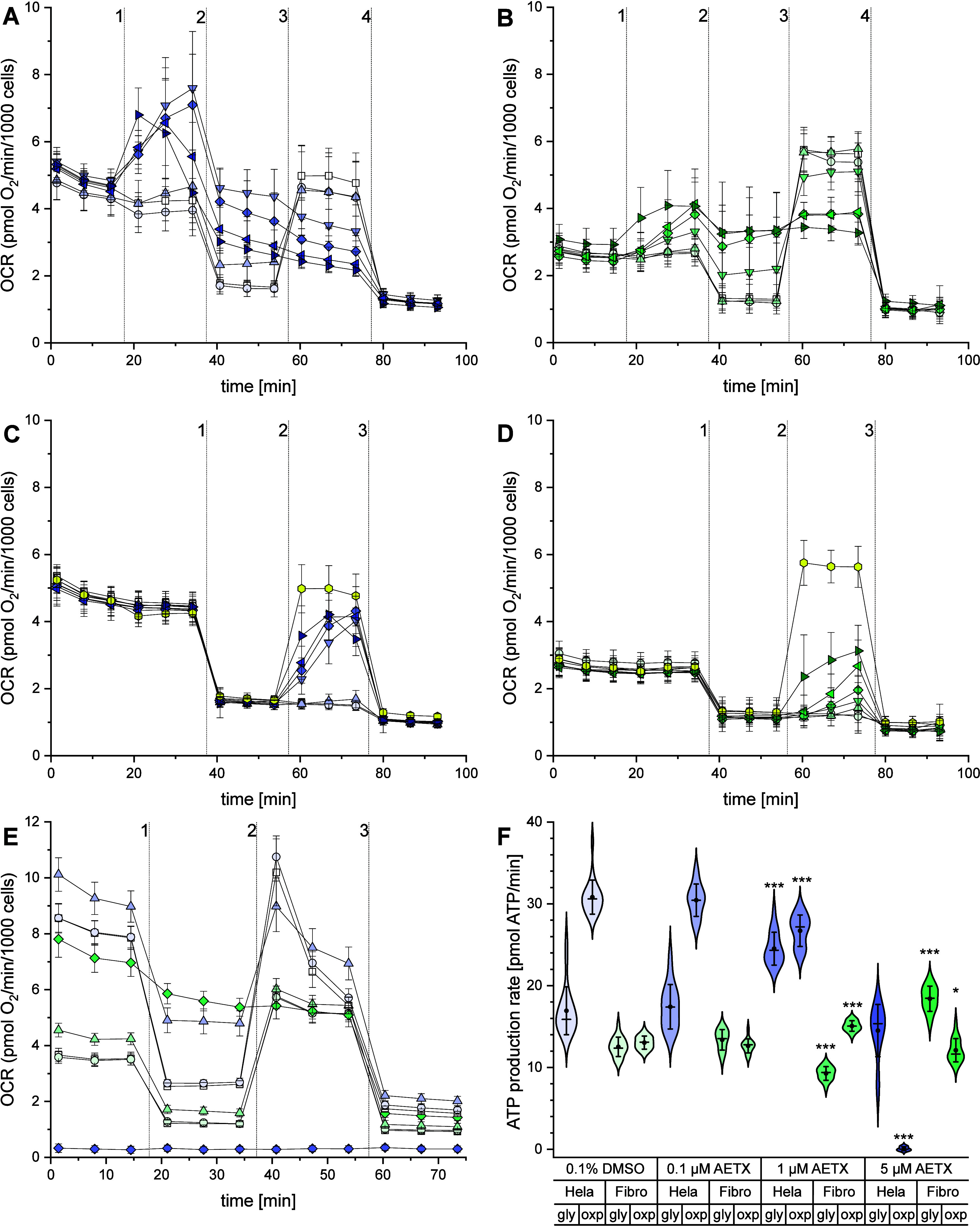
Influence of AETX on oxygen consumption and ATP production rates. (A–F) Violet: HeLa cells; green: fibroblasts. Increasing shades of color indicate increasing concentrations of AETX. Standard deviation (1×) indicated as whiskers. (A–E) Square: control (0.1% DMSO), circle: 0.1 μM AETX, triangle: 1 μM AETX, inverted triangle: 2 μM AETX, diamond: 5 μM AETX. (A, B) Left-pointing triangle: 10 μM, Right-pointing triangle: 30 μM AETX. (C, D) Yellow hexagon: FCCP. Oxygen consumption rate measurement of (A) HeLa cells or (B) fibroblasts after acute stimulation with AETX. Addition of 1: AETX, 2: oligomycin, 3: FCCP, 4: rotenone/antimycin A + Hoechst 33342. Oxygen consumption rate measurement of (C) HeLa cells or (D) fibroblasts after acute stimulation with AETX or FCCP subsequent to FoF1-ATPase inhibition. Addition of 1: oligomycin, 2: AETX or 2 μM FCCP, 3: rotenone/antimycin A + Hoechst 33342. (E) Oxygen consumption rate measurement of HeLa cells and fibroblasts after 24 h of treatment with AETX. Addition of 1: oligomycin, 2: FCCP, 3: rotenone/antimycin A + Hoechst 33342. (F) ATP production rate in HeLa cells and fibroblasts respective to the origin of ATP. Statistically significant difference to the control indicated with *p < 0.05 and ***p < 0.001, obtained with Mann–Whitney (HeLa cells) or Student's *t* test (fibroblasts). Mean indicated as black dot and the median as line.

Addition of AETX after inhibition of the F_o_F_1_-ATPase with oligomycin revealed a dose-dependent increase of OCR in both cell lines (Figure 4C,D), indicating an increased respiratory chain activity, which again agrees with an uncoupling effect of AETX.^16^ In comparison to that of FCCP, the rise in the level of the OCR induced by AETX was lower. This could be explained by the fact that we titrated FCCP in preliminary experiments to obtain the optimal response from each cell line, since uncouplers are known to have a bell-shaped effect curve on OCR,^46^ and that we did not test the respective optimal concentration of AETX.

The effects of rotenone and antimycin A, both added after AETX to the cells, were not influenced by any AETX treatment but were similar to the control throughout the experiments. This suggests that the targets of these compounds, complexes I and III of the respiratory chain, are not affected by AETX.

Uncoupling of the oxidative phosphorylation impacts the mitochondrial ATP production rate and interferes with the general energetic status of the cell.^17,47–49^ Therefore, we investigated the bioenergetic status of HeLa cells and fibroblasts after long-term incubation with AETX (Figure 4E,F). Both cell lines were incubated for 24 h with 0.1, 1, and 5 μM AETX before measuring the OCR and PER, followed by calculations of the ATP production rates according to Desousa et al.^17^ HeLa cells and fibroblasts treated with 0.1 μM AETX showed similar results compared to the control. At 1 μM AETX, the ATP production rate from oxidative phosphorylation decreased, while ATP generated from anaerobic glycolysis in HeLa cells was enhanced. In total, the ATP production rate increased compared to that of the control. A comparable effect was observed for fibroblasts after treatment with 5 μM AETX. These findings can be interpreted as a compensatory reaction of the cells responding to mitochondrial impairment. Treatment of HeLa cells with 5 μM AETX led to a decrease of total ATP production due to diminished oxidative phosphorylation. In contrast to treatment with 1 μM AETX, HeLa cells treated with 5 μM AETX did not adjust their glycolytic ATP production rate. The total ATP level, which we assessed in a luminescence-based assay, decreased accordingly (Figure S24). These results agree with our previous findings in the assays for cytotoxicity, as it might indicate that HeLa cells had already undergone cell cycle arrest, which is thought to be associated with lowered energy demand.^31^ However, affecting the electron transfer system may change the cytosolic NAD^+^/NADH+H^+^ ratio, which would alter glycolytic flux kinetics independently of cellular energy demand.^48^ Also, limitations in substrate transport due to diminished driving forces could contribute to this effect.^16,46^ On the other hand, fibroblasts that were incubated with 1 μM AETX showed an increased rate of ATP from oxidative phosphorylation, while glycolytic ATP generation was decreased. The experimentally determined total ATP level did not change compared to the control (Figure S24). As we could show that fibroblasts have a high spare respiratory capacity (Figure S25), it is plausible that these cells could productively use the stimulation with 1 μM AETX and regulate ATP production accordingly.

To orthogonally assess the interference of AETX with mitochondrial ATP generation, we hypothesized that the cytotoxicity of AETX should be exacerbated in cells capable of only oxidative phosphorylation. In order to test this hypothesis, HCT116 cells were cultured in medium either containing a high glucose concentration to force cells into anaerobic glycolysis or supplied only with pyruvate to force cells into oxidative phosphorylation. Indeed, we found an earlier onset of impairment (starting with 1 μM instead of 5 μM) and enhanced toxicity at 5 and 10 μM AETX in HCT116 cells cultivated in solely pyruvate containing medium when compared to the control and cells incubated in glucose containing medium (Figure S26). Furthermore, we determined the total ATP level in HeLa cells and fibroblasts cultured in pyruvate containing medium after treatment with AETX (Figure S24). Again, we observed an earlier onset of impairment in cancer cells (1 μM AETX in HeLa cells) and an exacerbated decrease of ATP level in both cell lines treated with 5 μM AETX. At this concentration, the notable drop of total ATP level is in accordance with the low ATP production rate from oxidative phosphorylation calculated for HeLa cells. These findings confirmed that AETX enhances the cellular need of glucose as an energy source to compensate for its effect on mitochondrial metabolism.

Having assessed oxygen consumption and ATP production rates, we strove to elucidate the effect of AETX on Δψ_m_ in both cell lines (Figure 3C,D). The Δψ_m_ was reduced in HeLa cells in all tested concentrations after acute stimulus and 2 h of incubation with AETX when compared to the control. In fibroblasts, Δψ_m_ rose when stimulated acutely with 5 and 10 μM AETX, increasing even further after 2 h of incubation, while cells treated with 0.1 and 1 μM displayed a reduced Δψ_m_. Again, the observed differences between both cell lines in reaction to AETX can be attributed to the lower maximum respiratory capacity of HeLa cells compared to that of fibroblasts. The maximum respiratory capacity is utilized to strengthen the Δψ_m_ consuming NADH-linked substrates, such as pyruvate, malate, or succinate.^50^ After 24 h of treatment, however, it can be assumed that the substrate availability is reduced. Thus, the fibroblasts might no longer be able to fuel the increased respiratory chain activity, and therefore the Δψ_m_ decreases accordingly. Indeed, after 24 h of treatment, all concentrations of AETX resulted in reduced Δψ_m_ in fibroblasts. This effect, determined in both cell lines, is characteristic for uncouplers.^49,50^ In addition, a low Δψ_m_ is associated with cell cycle arrest in the early G_1_ phase,^31^ which we indeed observed as described above.

Furthermore, the formation of reactive oxygen species (ROS) can be influenced by the interference of uncouplers with the pmf.^51,52^ We deliberately chose 2′,7′-dichlorfluorescein-diacetate (DCF-DA) as a fluorescent probe to simultaneously assess the cellular unspecific ROS level and mobilization of cytochrome c from complex VI of the respiratory chain as an indication of mitochondrial damage.^53^ We detected only a slight reduction of the DCF-DA signal after incubation of HeLa cells and fibroblasts with AETX (Figure S27), suggesting that complex IV of the respiratory chain remains intact after treatment with AETX and that ROS formation is largely unaffected in the first 2 h after AETX treatment but might be slightly reduced after 24 h of incubation.

### AETX Acts as a Protonophore on Artificial Lipid Bilayers

In order to assess the capability of AETX to translocate protons across membranes in a protein-independent manner, we measured the conductance of a protein-free, artificial planar lipid bilayer separating two chambers containing aqueous solutions in the presence of AETX or FCCP as a positive control. First, we conducted the experiment with solutions of pH 7 on both sides of the membrane. We detected voltage-dependent currents for both compounds (Figure 5, white symbols). After the addition of hydrochloric acid to one of the chambers to obtain an aqueous solution of pH 3, the electric currents increased in a voltage-dependent manner, again for both compounds (Figure 5, colored symbols). Comparing AETX to FCCP, we found similar conductance value increases (Figure S28). Our data demonstrate that AETX can shuttle protons across a membrane.

**Figure 5:**
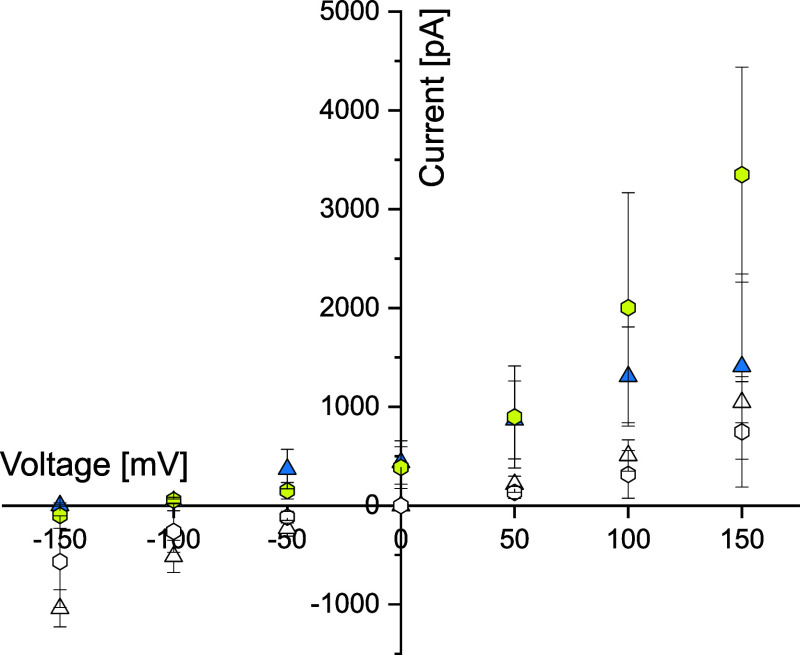
Current–voltage plot displaying translocation of protons across a protein-free artificial planar lipid bilayer. Triangle: 1 μM AETX; hexagon: 1 μM FCCP. Yellow/blue: measurement in water with pH 3 on the amplifier side. White: control measurement in HEPES-buffered solution at pH 7. Whiskers indicate standard deviation (1×).

However, it has been reported that uncouplers might also interact with mitochondrial proteins to exert their effect on oxidative phosphorylation. Kotova and Antonenko discussed that the effective concentration of certain uncouplers differs when tested on artificial lipid bilayers or in intact mitochondria. They concluded that active interaction of uncouplers with proton pumps or other proteins of the inner mitochondrial membrane might occur.^46^ Indeed, it has been shown that, e.g., FCCP or DNP increases the proton permeability of the mitochondrial membrane via induction of the ADP/ATP carrier and uncoupling protein 1 activity.^54^ Our study is limited in this regard, as we can yet neither exclude nor suggest any additional interaction of AETX with mitochondrial proteins. However, the artificial lipid bilayer membrane assay demonstrated the capability of AETX to transport protons across membranes, confirming the underlying mechanism of action of AETX as a weakly acidic uncoupler.

If AETX is indeed a weakly acidic uncoupler, then its slightly acidic indole nitrogen should be crucial for its function as a protonophore, as it is known that blocking the acidic group of uncouplers, e.g., by methylation, abolishes their activity on the respiratory chain.^55^ We further hypothesized that removal of the electron-withdrawing nitrile group should diminish its activity since it should decrease NH acidity. To test these hypotheses, we synthesized *N*-methyl-AETX (m-AETX) by methylation of AETX using dimethyl sulfate. Desnitrile-AETX (dn-AETX) was isolated from the AETX-producing cyanobacterium *A. hydrillicola*, as described before.^25^

Comparing the cytotoxicity of AETX and its two derivatives showed that after 24 h of incubation, m-AETX indeed was inactive at all tested concentrations (up to 10 μM), while dn-AETX, as expected, was less cytotoxic than AETX (Figure 6A). The importance of the secondary amine and the nitrile group for the function of AETX as protonophore and, hence, uncoupler also became obvious when we compared the acute dissipation of the Δψ_m_ in HeLa cells induced by AETX, m-AETX, and dn-AETX: While treatment with AETX led to immediate and concentration-dependent dissipation of Δψ_m_, both derivatives rather hyperpolarized the mitochondrial membrane independent of the treatment concentration (Figure 6B).

**Figure 6:**
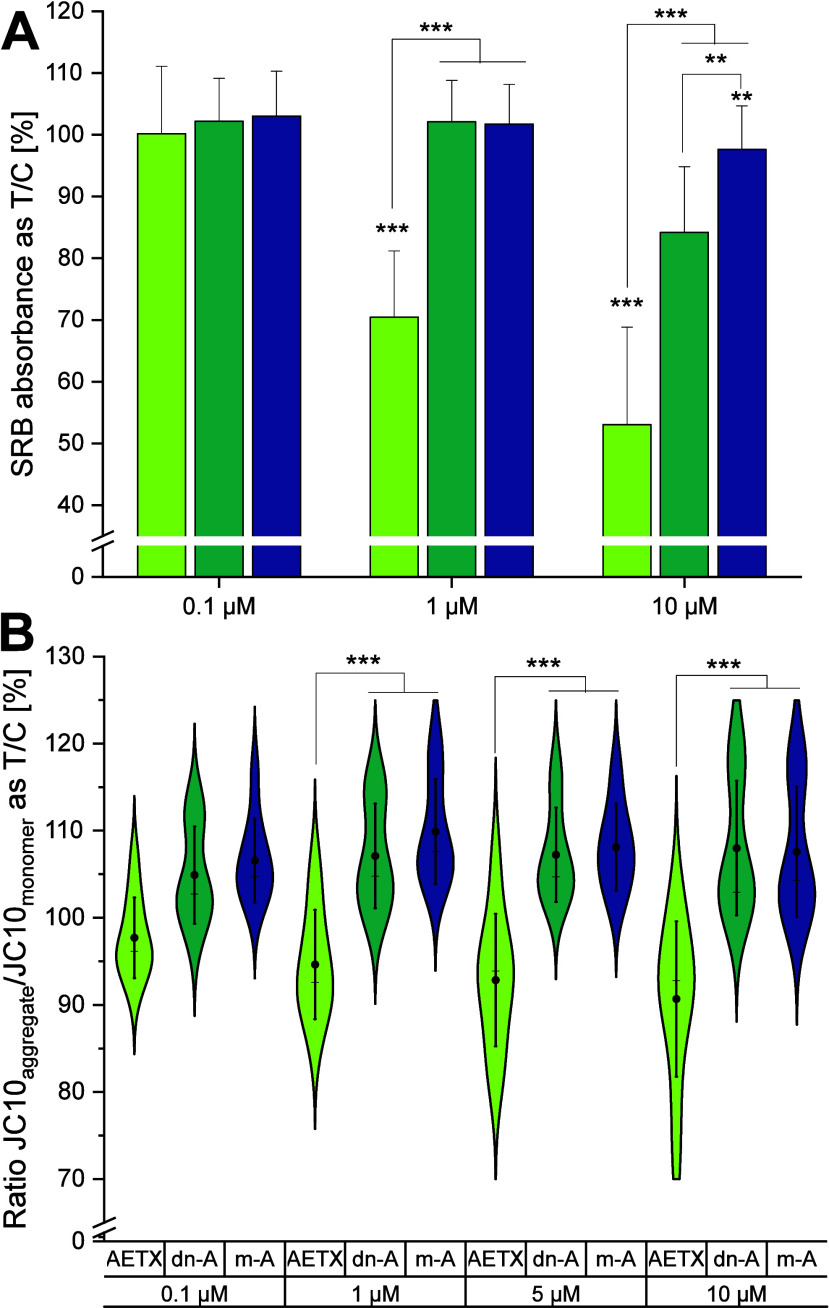
Comparison of the effect of AETX, *N*-methyl-AETX (m-AETX, m-A), and desnitrile-AETX (dn-AETX, dn-A) on overall cell viability and mitochondrial membrane potential. (A, B) Light green: AETX, green: dn-AETX, blue: m-AETX. Standard deviation (1×) indicated as whiskers. (A) Cytotoxicity assay based on protein content (sulforhodamine B absorbance) in HCT116 cells normalized to the control. Statistically significant difference to the control indicated with **p < 0.01 and ***p < 0.001, obtained with the Mann–Whitney test. (B) Mitochondrial membrane potential as the ratio of JC10 aggregate to JC10 monomer in treated HeLa cells normalized to the control. Mean indicated as black dot and the median as line. Statistically significant difference to treatment with AETX indicated with ***p < 0.001, obtained with Student's *t* test.

## Conclusions

We found that AETX acts as an uncoupler of oxidative phosphorylation. We assume that the cytostatic and bacteriostatic effects we initially observed are due to a lack of ATP in the affected cells. This could also explain the observed morphological changes of *B. subtilis* from rod to spheric shape at concentrations below the MIC, as the elongation but not the division of *B. subtilis* is dependent on the ATP-binding cassette transporter-like complex FtsEX.^56^ For eukaryotic cells, perturbations in cellular energy homeostasis resulting in elevated AMP:ATP ratios are a known switch on the canonical AMP-dependent mechanism of AMP-activated protein kinase (AMPK) activation. Among other downstream effects, activated AMPK leads to enhanced mitophagy and cell cycle arrest in the G_1_ phase.^57^ The reduced ATP production rate, the reduced TOM20 signals of mitochondria, and the cell cycle arrest in the G_1_ phase we observed in our assays suggest that AMPK activation is a downstream effect of AETX-induced uncoupling. Furthermore, AMPK activation in neurons induces Kv2.1 channel opening.^58^ Prolonged imbalances in neuronal ion homeostasis altering osmotic conditions in the brain could lead to the formation of the large intramyelinic vacuoles observed in VM.^59,60^ In addition, deficiency of the respiratory chain has been linked to myelin splitting at the minor dense lines like observed in animals suffering from AETX-induced VM.^2,61^ So far, however, there is no direct evidence for the effect of AETX on myelinating cells or other targets mentioned above, so these considerations need to be examined in more detail in future studies. The efficacy of AETX shown in the diversity of models used in our study is reflected by the broad range of affected animals, i.e., comprising taxa with and without myelin, emphasizing mitochondria as the universal primary target of AETX intoxication.

## Supplementary Material





## Notes

Metabolomics experiments (XLSX)
Detailed discussion of the structure confirmation of m-AETX; biological assays, HPLC-UV chromatograms of AETX and derivatives, MS data, and NMR spectra of m-AETX (PDF)

## ▲

Stephen F. Donovan is retired.

## ABBREVIATIONS

AETX: Aetokthonotoxin
AMP: adenosine monophosphate
AMPK: AMP-activated protein kinase
CCCP: [(3-chlorophenyl)­hydrazono]­malononitrile
CLSI: Clinical and Laboratory Standards Institute
DCF-DA: 2′,7′-dichlorfluorescein-diacetate
DNP: 2,4-dinitrophenol
EC_50_: half-maximal effect concentration
FBS: fetal bovine serum
FCCP: 2-[[4-(trifluoromethoxy)­phenyl]­hydrazinylidene]­propanedinitrile
GDH: glutamate dehydrogenase
GI_50_: half-maximal growth inhibition
LC_50_: half-maximal lethal concentration
MBC: minimal bactericidal concentration
MIC: minimal inhibitory concentration
MoA: mode of action
NCI: National Cancer Institute
Δψ_m_: mitochondrial membrane potential
OCR: oxygen consumption rate
PER: proton efflux rate
ΔpH: proton gradient
pmf: proton motive force
RAA: rotenone/antimycin A
ROS: reactive oxygen species
TGI: total growth inhibition
TOM20: mitochondrial translocase of the outer membrane
SAR: structure–activity relationship
SRB: sulforhodamine B
VM: vacuolar myelinopathy
