# Supplementary material for: Aetokthonotoxin, the Causative Agent of Vacuolar Myelinopathy, Uncouples Oxidative Phosphorylation due to Protonophore Activity
Source: Chem Res Toxicol. 2025 Jul 25;38(9):1495–508. doi: 10.1021/acs.chemrestox.5c00147 (PMC12442230; doi:10.1021/acs.chemrestox.5c00147)
Supplement: Supplementary file 2 [file tx5c00147_si_002.pdf]

Supporting Information for

Aetokthonotoxin, the causative agent of vacuolar myelinopathy, uncouples oxidative phosphorylation due to protonophore activity

Valerie I. C. Rebhahn[°,‖], Mohamad Saoud[†], Mathias Winterhalter[‡], Franziska Schanbacher[°], Maximilian Jobst[§,¶], Rebeca Ruiz[#], Alexander Sonntag[°], Johannes Kollatz[°,†], Rieke Sprengel[°], Stephen F. Donovan[%], Giorgia Del Favero[§], Robert Rennert[†], Timo H. J. Niedermeyer[*,°,‖]

[*] Corresponding author: Prof. Dr. Timo H. J. Niedermeyer; email: timo.niedermeyer@fu-berlin.de
[°] Institute of Pharmacy, Pharmaceutical Biology, Freie Universität Berlin, 14195 Berlin, Germany
[†] Department of Bioorganic Chemistry, Leibniz Institute of Plant Biochemistry, 06120 Halle (Saale), Germany
[‡] School of Science, Constructor University Bremen gGmbH, 28759 Bremen, Germany, and Center for Hybrid Nanostructures, Universität Hamburg, 22761 Hamburg, Germany
[§] Department of Food Chemistry and Toxicology, and Core Facility Multimodal Imaging, Faculty of Chemistry, University of Vienna, 1090, Vienna, Austria.
[¶] University of Vienna, Vienna Doctoral School in Chemistry (DoSChem), 1090 Vienna, Austria
[#] Pion Inc., Forest Row Business Park, Forest Row RH18 5DW, United Kingdom
[%] Retired
[‖] Part of this work was done at the Department of Pharmaceutical Biology/Pharmacognosy, Institute of Pharmacy, Martin Luther University Halle-Wittenberg, 06120 Halle (Saale), Germany

Table of Contents

	Page
**SUPPLEMENTARY TEXT**	
Structure confirmation of *N*-methyl-AETX (m-AETX)	2
**SUPPLEMENTARY FIGURES**	
Microbiology	3
Cell biology – cytotoxicity	4
Metabolomics	12
Chemistry – pK_a and logP	15
Cell biology – Seahorse experiments I	18
Cell biology – total ATP level	24
Cell biology – Seahorse experiments II	25
Cell biology – enhanced cytotoxicity	26
Cell biology – ROS	27
Biophysics – artificial lipid bilayer conductance	28
Chemistry – purity of AETX, dn-AETX, m-AETX	29
Chemistry – structure confirmation of m-AETX	29

SUPPLEMENTARY TEXT

Structure confirmation of *N*-methyl-AETX (m-AETX). The structure of m-AETX, and thus the successful *N*-methylation of AETX, was confirmed by 1D and 2D NMR spectroscopy and high-resolution mass spectrometry experiments based on the structure elucidation of AETX conducted by Breinlinger *et al.* [1]. HRMS analysis resulted in a $[M+H]^+$ ion at m/z 667.6651, from which the molecular formula $C_{18}H_9N_3{}^{79}Br_2{}^{81}Br_3$ was calculated (calc. 667.6652, Δ 0.1 ppm). The presence of five bromo substituents in the molecule was also obvious from the observed isotope pattern. In contrast to AETX, which can only be ionized efficiently in negative ionization mode, m-AETX showed poor ionization efficiency, with ion signals detected exclusively in positive mode. The ^1H spectrum of m-AETX showed five distinct signals with chemical shifts in the aromatic region corresponding to the five protons of the two indole subunits. The chemical shifts (δ, in ppm) of the protons, the integration of these signals, and the coupling constants (J, measured in Hz) were as follows: δ 8.06 (d, J= 1.8 Hz, 1H), C-4'; δ 7.96 ppm (d, J= 1.8 Hz, 1H), C-6'; δ 7.79 ppm (dd, J= 1.8 Hz, J= 0.5 Hz, 1H), C-4; δ 7.52 ppm (dd, J= 8.8 Hz, J= 1.9 Hz, 1H), C-6; δ 7.40 ppm (dd, J= 8.8 Hz, J= 0.5 Hz, 1H), C-7, agreeing well with the data of AETX [1]. An additional proton signal in the spectrum indicated the presence of a methyl group at δ 3.77 (s, 3H). In addition to the previously reported long-range correlations in AETX (key HMBC correlations are shown in Fig. S29, two additional long-range $^3J_{CH}$ couplings were observed in the HMBC spectrum: a correlation between the methyl group (*N*-methyl) and C-7a', and a correlation between the methyl group and C-2'. Based on these data, the successful *N*-methylation of AETX was confirmed. Fig. S31 shows the HRMS spectrum, Fig. S32, Fig. S33 and Fig. S34 show the ^1H, ^{13}C-HMBC and ^{13}C-HSQC NMR spectra of m-AETX at 700 MHz.

SUPPLEMENTARY REFERENCES

(1) Breinlinger, S.; Phillips, T. J.; Haram, B. N.; Mareš, J.; Martínez Yerena, J. A.; Hrouzek, P.; Sobotka, R.; Henderson, W. M.; Schmieder, P.; Williams, S. M.; Lauderdale, J. D.; Wilde, H. D.; Gerrin, W.; Kust, A.; Washington, J. W.; Wagner, C.; Geier, B.; Liebeke, M.; Enke, H.; Niedermeyer, T. H. J.; Wilde, S. B. Hunting the eagle killer: A cyanobacterial neurotoxin causes vacuolar myelinopathy. *Science* **2021**, *371* (6536). DOI: 10.1126/science.aax9050.

SUPPLEMENTARY FIGURES

Microbiology

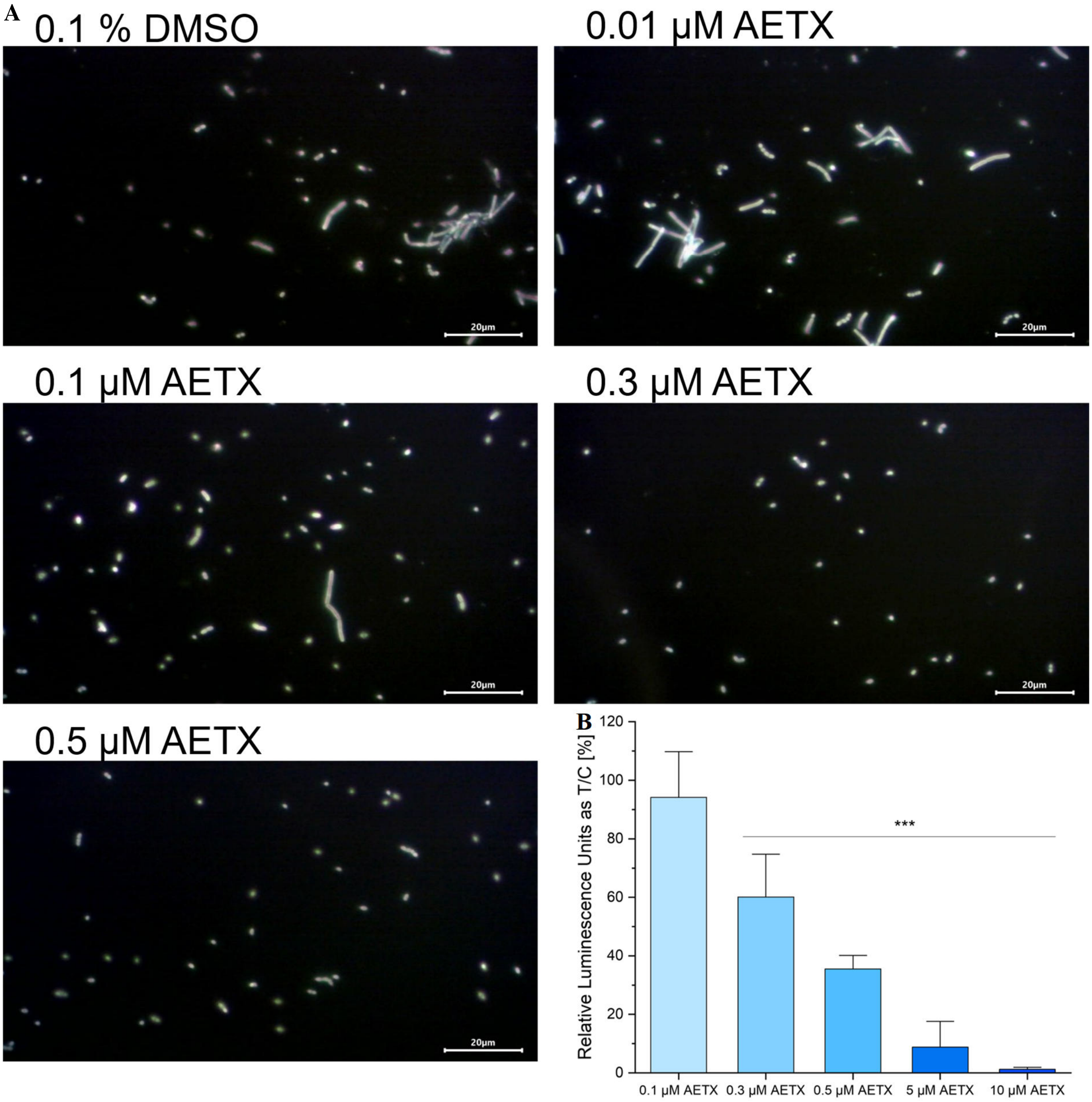

Fig. S1: Effects of AETX on *B. subtilis*. **(A)** Darkfield microscopy images of morphological changes from rod to spheric shape of *B. subtilis* after 24 h incubation with 0.1% DMSO or 0.01, 0.1, 0.3, and 0.5 µM AETX. Scale bar 20 µm. (**B**) Relative luminescence units showing the amount of ATP in *B. subtilis* compared to the control (0.1% DMSO) after 24 h incubation with 0.1, 0.3, 0.5, 5, and 10 µM AETX. Boxes present mean with 1X standard devaition as whisker. Statistically significant difference to the control indicated with *** $p < 0.001$, obtained with Mann-Whitney test. Data based on three biological replicates.

Cell biology – cytotoxicity

A

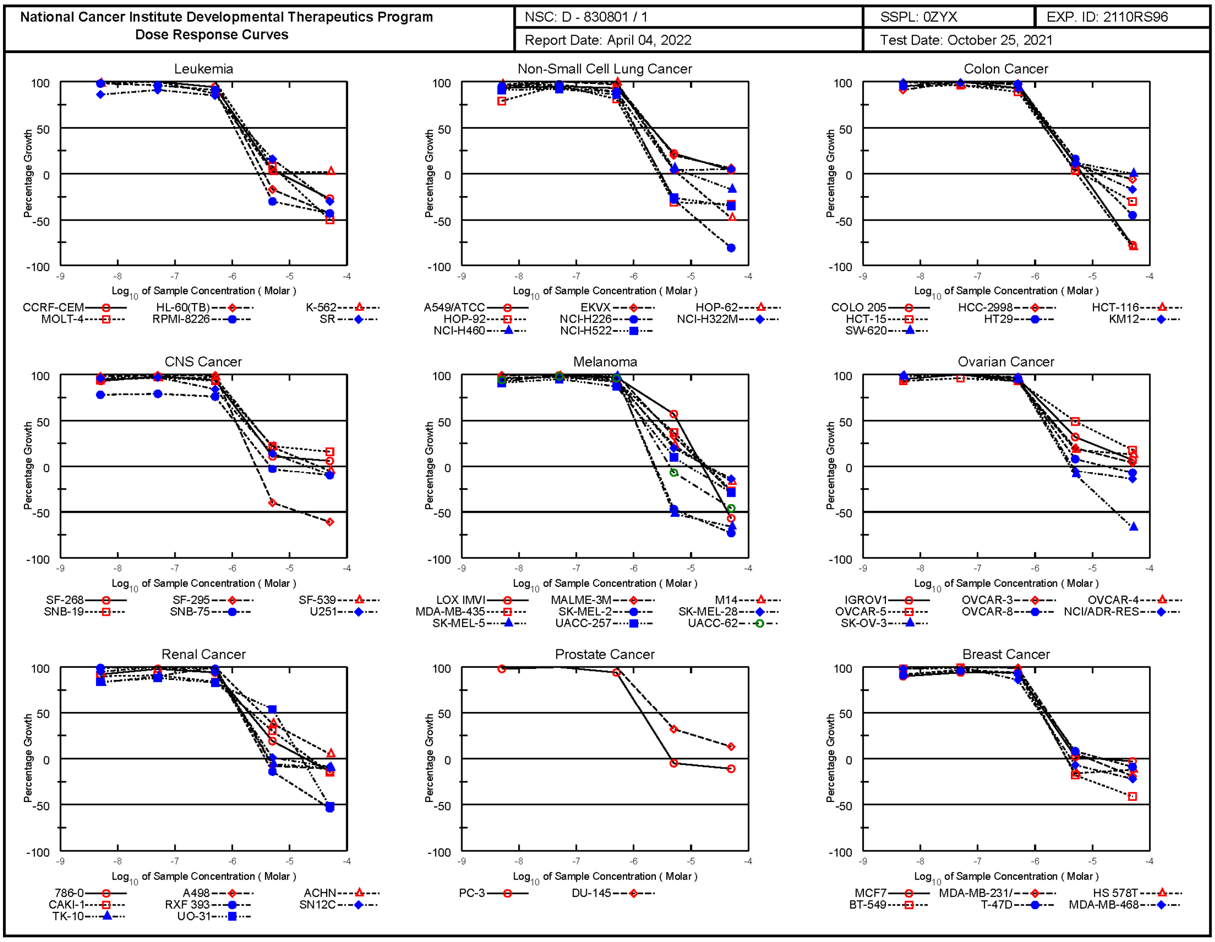

B

National Cancer Institute Developmental Therapeutics Program
In-Vitro Testing Results

NSC : D - 830801 / 1	Experiment ID : 2110RS96	Test Type : 08	Units : Molar
Report Date : April 04, 2022	Test Date : October 25, 2021	QNS :	MC :
COMI : AETX	Stain Reagent : SRB Dual-Pass Related	SSPL : 0ZYX	

Panel/Cell Line	Time Zero	Ctrl	Mean Optical Densities -8.3	-7.3	-6.3	-5.3	-4.3	Percent Growth -8.3	-7.3	-6.3	-5.3	-4.3	GI50	TGI	LC50
**Leukemia**															
CCRF-CEM	0.499	2.474	2.658	2.854	2.352	0.573	0.363	109	119	94	4	-27	1.53E-6	6.59E-6	> 5.00E-5
HL-60(TB)	0.561	2.401	2.393	2.538	2.590	0.468	0.319	100	107	110	-17	-43	1.49E-6	3.70E-6	> 5.00E-5
K-562	0.184	2.006	1.966	2.071	1.772	0.229	0.230	98	104	87	2	2	1.37E-6	> 5.00E-5	> 5.00E-5
MOLT-4	0.573	2.847	2.867	3.032	2.846	0.765	0.289	101	108	100	8	-50	1.76E-6	6.99E-6	> 5.00E-5
RPMI-8226	0.997	3.005	2.963	2.935	2.819	0.701	0.572	98	96	91	-30	-43	1.09E-6	2.83E-6	> 5.00E-5
SR	0.550	2.546	2.265	2.361	2.255	0.865	0.385	86	91	85	16	-30	1.61E-6	1.10E-5	> 5.00E-5
**Non-Small Cell Lung Cancer**															
A549/ATCC	0.872	2.984	2.828	2.880	2.830	1.339	0.966	93	95	93	22	4	2.01E-6	> 5.00E-5	> 5.00E-5
EKVX	0.803	2.244	2.172	2.243	2.207	1.087	0.886	95	100	97	20	6	2.04E-6	> 5.00E-5	> 5.00E-5
HOP-62	0.902	2.681	2.633	2.696	2.671	0.977	0.473	97	101	99	4	-48	1.65E-6	6.03E-6	> 5.00E-5
HOP-92	1.688	2.213	2.100	2.191	2.112	1.171	1.130	79	96	81	-31	-33	9.45E-7	2.65E-6	> 5.00E-5
NCI-H226	1.682	2.750	2.692	2.723	2.633	1.211	0.317	95	97	89	-28	-81	1.08E-6	2.88E-6	1.30E-5
NCI-H322M	0.792	2.257	2.164	2.153	2.100	0.857	0.870	94	93	89	4	5	1.45E-6	> 5.00E-5	> 5.00E-5
NCI-H460	0.230	2.330	2.395	2.419	2.374	0.363	0.192	103	104	102	6	-17	1.75E-6	9.46E-6	> 5.00E-5
NCI-H522	1.048	2.901	2.741	2.753	2.649	0.773	0.685	91	92	86	-26	-35	1.05E-6	2.92E-6	> 5.00E-5
**Colon Cancer**															
COLO 205	0.480	2.117	2.111	2.165	1.995	0.657	0.105	100	103	93	11	-78	1.66E-6	6.62E-6	2.41E-5
HCC-2998	0.737	2.487	2.328	2.532	2.447	0.918	0.693	91	103	98	10	-6	1.76E-6	2.14E-5	> 5.00E-5
HCT-116	0.228	2.316	2.348	2.239	2.168	0.264	0.046	102	96	93	2	-80	1.48E-6	5.25E-6	2.15E-5
HCT-15	0.382	2.537	2.438	2.454	2.311	0.475	0.268	95	96	89	4	-30	1.45E-6	6.68E-6	> 5.00E-5
HT29	0.369	2.401	2.300	2.420	2.344	0.697	0.204	95	101	97	16	-45	1.91E-6	9.20E-6	> 5.00E-5
KM12	0.696	3.217	3.182	3.189	3.138	0.969	0.578	99	99	97	11	-17	1.75E-6	1.23E-5	> 5.00E-5
SW-620	0.267	1.981	2.000	2.072	1.863	0.467	0.272	101	105	93	12	0	1.69E-6	> 5.00E-5	> 5.00E-5
**CNS Cancer**															
SF-268	0.898	2.630	2.502	2.595	2.531	1.088	1.000	93	98	94	11	6	1.70E-6	> 5.00E-5	> 5.00E-5
SF-295	0.951	2.656	2.618	2.642	2.633	0.575	0.371	98	99	99	-40	-61	1.12E-6	2.59E-6	1.53E-5
SF-539	0.706	2.408	2.330	2.348	2.364	1.059	0.669	95	96	97	21	-5	2.08E-6	3.14E-5	> 5.00E-5
SNB-19	0.553	1.944	1.861	1.901	1.850	0.863	0.774	94	97	93	22	16	2.03E-6	> 5.00E-5	> 5.00E-5
SNB-75	1.689	2.686	2.464	2.475	2.445	1.646	1.517	78	79	76	-3	-10	1.07E-6	4.64E-6	> 5.00E-5
U251	0.706	2.843	2.747	2.778	2.498	1.004	0.639	96	97	84	14	-9	1.52E-6	1.97E-5	> 5.00E-5
**Melanoma**															
LOX IMVI	0.320	1.903	1.901	1.894	1.856	1.219	0.137	100	99	97	57	-57	5.74E-6	1.57E-5	4.31E-5
MALME-3M	0.621	1.226	1.222	1.233	1.232	0.820	0.445	99	101	101	33	-28	2.80E-6	1.72E-5	> 5.00E-5
M14	0.560	2.343	2.264	2.316	2.198	0.963	0.468	96	98	92	23	-17	2.01E-6	1.89E-5	> 5.00E-5
MDA-MB-435	0.499	2.308	2.241	2.246	2.188	1.167	0.366	96	97	93	37	-27	2.93E-6	1.90E-5	> 5.00E-5
SK-MEL-2	1.722	2.753	2.667	2.760	2.724	0.911	0.470	92	101	97	-47	-73	1.06E-6	2.36E-6	6.49E-6
SK-MEL-28	0.625	1.992	2.016	2.036	1.909	0.895	0.536	102	103	94	20	-14	1.95E-6	1.91E-5	> 5.00E-5
SK-MEL-5	0.912	3.297	3.309	3.296	3.245	0.434	0.311	101	100	98	-52	-66	1.04E-6	2.24E-6	4.81E-6
UACC-257	1.294	2.910	2.762	2.827	2.704	1.455	0.914	91	95	87	10	-29	1.52E-6	8.95E-6	> 5.00E-5
UACC-62	0.837	2.868	2.748	2.818	2.794	0.776	0.454	94	98	96	-7	-46	1.40E-6	4.25E-6	> 5.00E-5
**Ovarian Cancer**															
IGROV1	0.520	2.317	2.232	2.316	2.240	1.097	0.643	95	100	96	32	7	2.62E-6	> 5.00E-5	> 5.00E-5
OVCAR-3	0.601	2.068	2.110	2.112	1.964	0.895	0.660	103	103	93	20	4	1.94E-6	> 5.00E-5	> 5.00E-5
OVCAR-4	0.656	1.752	1.696	1.758	1.662	0.851	0.794	95	101	92	18	13	1.83E-6	> 5.00E-5	> 5.00E-5
OVCAR-5	0.703	1.878	1.802	1.831	1.796	1.282	0.915	93	96	93	49	18	4.81E-6	> 5.00E-5	> 5.00E-5
OVCAR-8	0.308	1.588	1.591	1.598	1.523	0.407	0.288	100	101	95	8	-7	1.64E-6	1.72E-5	> 5.00E-5
NCI/ADR-RES	0.581	1.996	1.976	2.070	1.957	0.555	0.497	99	105	97	-5	-14	1.45E-6	4.51E-6	> 5.00E-5
SK-OV-3	1.170	2.256	2.230	2.288	2.207	1.068	0.384	98	103	95	-9	-67	1.36E-6	4.12E-6	2.54E-5
**Renal Cancer**															
786-0	0.478	2.335	2.178	2.300	2.221	0.835	0.411	92	98	94	19	-14	1.93E-6	1.89E-5	> 5.00E-5
A498	1.867	2.520	2.543	2.608	2.566	1.712	1.665	103	113	107	-8	-11	1.56E-6	4.23E-6	> 5.00E-5
ACHN	0.260	1.404	1.423	1.455	1.404	0.698	0.312	102	104	100	38	5	3.23E-6	> 5.00E-5	> 5.00E-5
CAKI-1	0.540	1.940	1.799	1.820	1.710	0.956	0.459	90	91	84	30	-15	2.10E-6	2.30E-5	> 5.00E-5
RXF 393	0.825	1.459	1.450	1.524	1.444	0.706	0.380	99	110	98	-14	-54	1.33E-6	3.72E-6	3.96E-5
SN12C	0.572	2.204	2.124	2.204	2.100	0.590	0.522	95	100	94	1	-9	1.48E-6	6.43E-6	> 5.00E-5
TK-10	1.383	2.246	2.101	2.162	2.256	1.299	1.246	83	90	101	-6	-10	1.50E-6	4.39E-6	> 5.00E-5
UO-31	0.610	2.003	1.779	1.839	1.766	1.362	0.292	84	88	83	54	-52	5.45E-6	1.61E-5	4.77E-5
**Prostate Cancer**															
PC-3	0.625	2.607	2.575	2.672	2.498	0.594	0.558	98	103	94	-5	-11	1.40E-6	4.45E-6	> 5.00E-5
DU-145	0.442	2.024	2.095	2.146	2.062	0.946	0.641	104	108	102	32	13	2.76E-6	> 5.00E-5	> 5.00E-5
**Breast Cancer**															
MCF7	0.461	2.309	2.134	2.205	2.201	0.502	0.445	90	94	94	2	-3	1.51E-6	1.23E-5	> 5.00E-5
MDA-MB-231/ATCC	0.540	1.468	1.476	1.520	1.457	0.593	0.439	101	106	99	6	-19	1.67E-6	8.52E-6	> 5.00E-5
HS 578T	1.734	2.745	2.665	2.720	2.685	1.454	1.521	92	97	94	-16	-12	1.25E-6	3.57E-6	> 5.00E-5
BT-549	1.658	2.960	2.940	2.948	2.974	1.354	0.985	98	99	101	-18	-41	1.34E-6	3.51E-6	> 5.00E-5
T-47D	0.966	2.279	2.161	2.221	2.193	1.069	0.876	91	96	93	8	-9	1.61E-6	1.43E-5	> 5.00E-5
MDA-MB-468	0.790	1.327	1.317	1.347	1.252	0.736	0.613	98	104	86	-7	-22	1.22E-6	4.21E-6	> 5.00E-5

(Log10 Concentration)

Fig. S2: Cytotoxicity data derived from the NCI-60 panel of the National Cancer Institute (NCI) Developmental Therapeutics Program (five-dose-SRB-assay, 5 nM to 50 µM). **(A)** Effect of AETX on cell growth in % on various cancer cell lines. **(B)** Individual data for every cancer cell line. Results are summarized according to cell line origin.GI50: growth inhibition 50%, TGI: total growth inhibition, LC50: lethal concentration 50%.

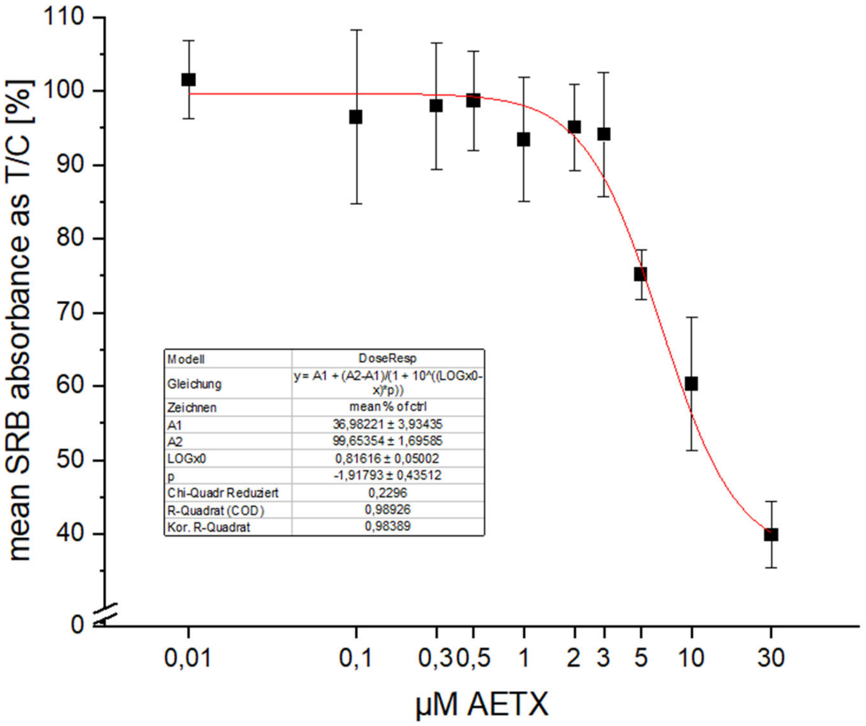

Fig. S3: Mean SRB absorbance of AETX-treated HeLa cells normalized to the control. Whiskers indicate standard deviation. Red line: calculated fit. EC_{50} 6.5 µM.

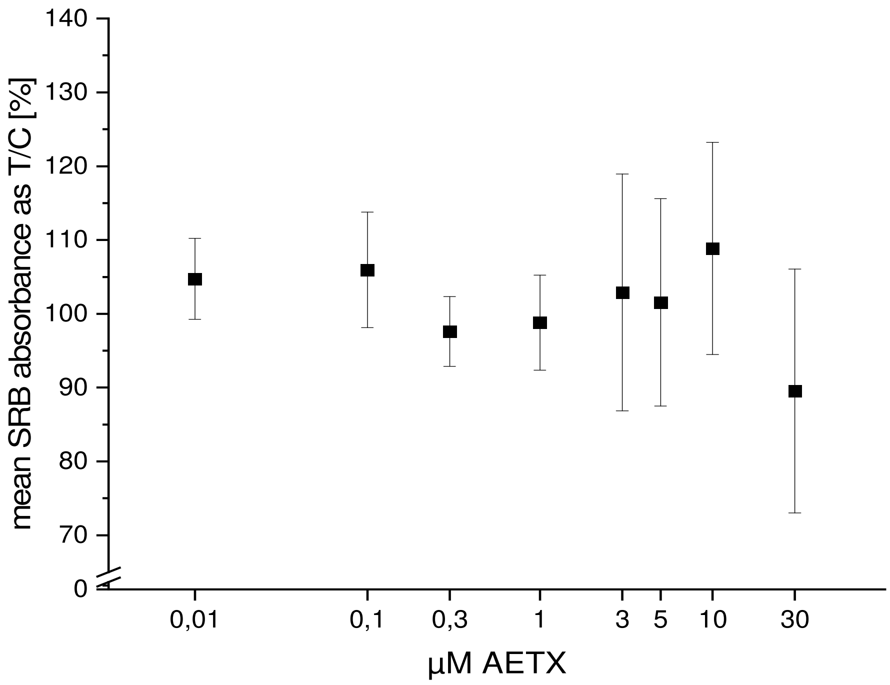

Fig. S4: Mean SRB absorbance of AETX-treated fibroblasts normalized to the control. Whiskers indicate standard deviation. A fit could not be calculated. EC_{50} > 30 µM.

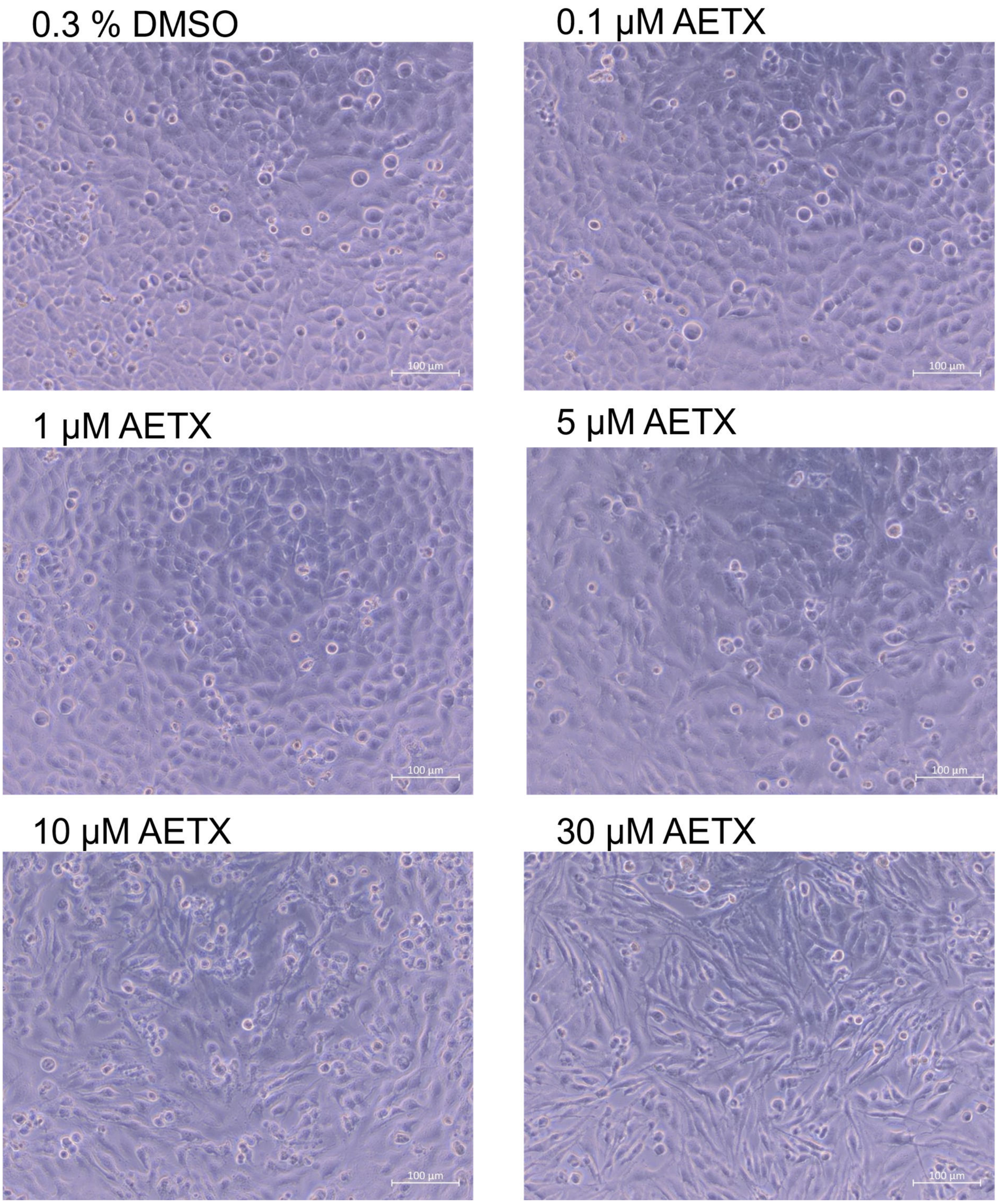

Fig. S5: Phase contrast microscopy images of morphological changes of HeLa cells incubated with 0.3% DMSO, 0.1, 1, 5, 10, and 30 µM AETX for 24 h. Scale bar 100 µm.

A

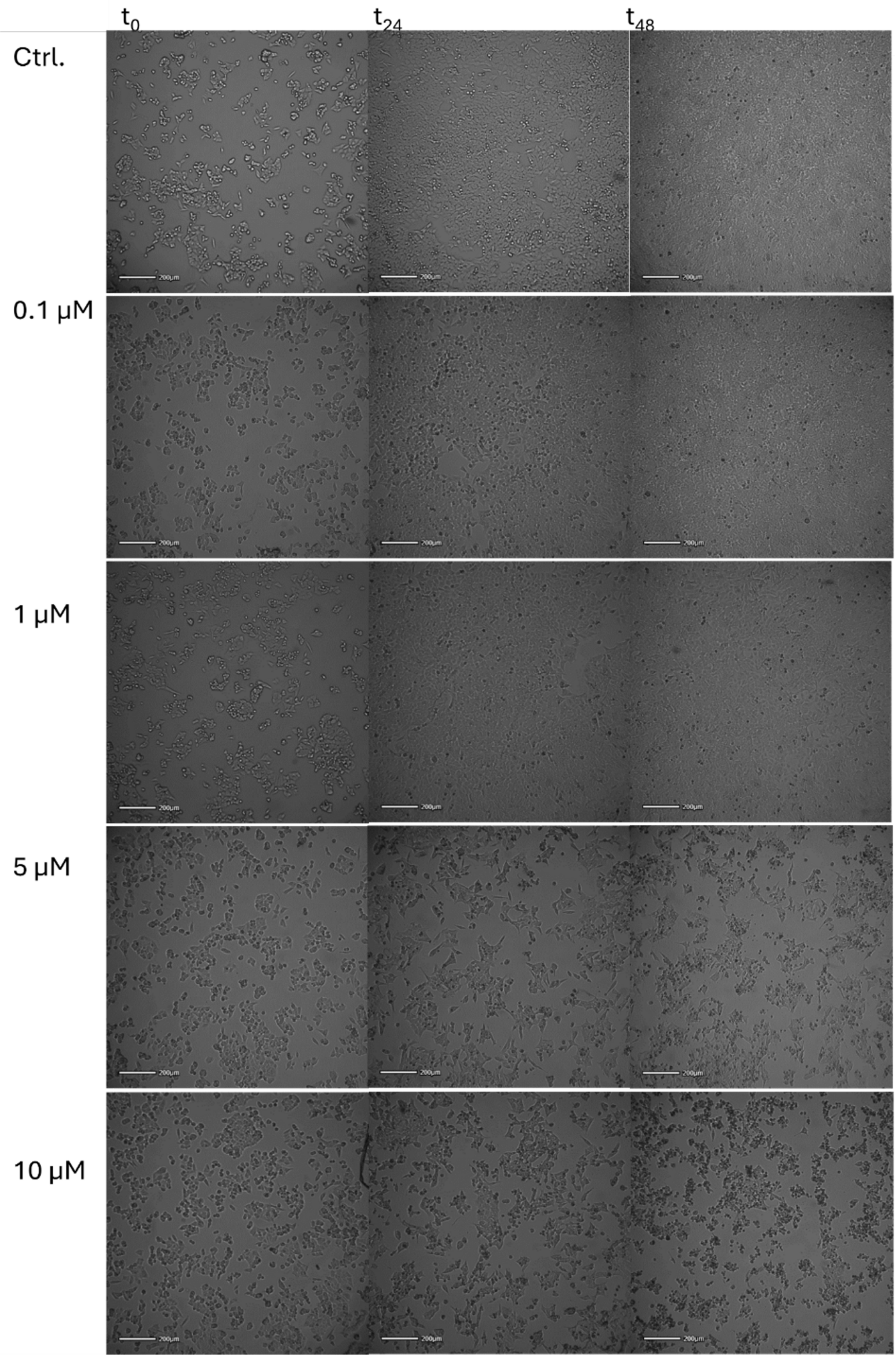

B

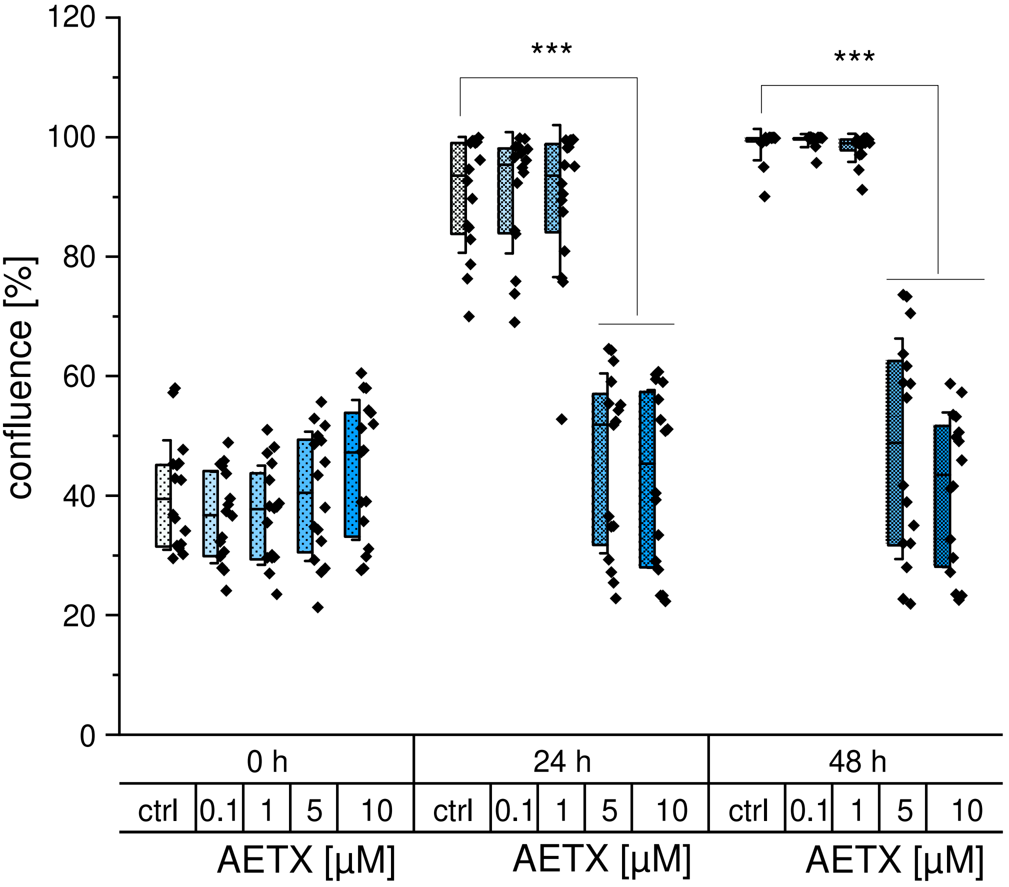

Fig. S6: Effect of AETX on HCT116 cell confluency. **(A)** Brightfield microscopy images showing confluency of AETX-treated HCT116 cells directly (t0), 24 h (t24) and 48 h (t48) after treatment. Scale bar 200 µm. **(B)** Confluency of AETX-treated HCT116 cells at different timepoints after treatment in % of covered area acquired from brightfield microscopy images. Cells were treated with 0.1% DMSO (ctrl), 0.1, 1, 5, or 10 µM AETX. Statistically significant difference to the control indicated with *** $p < 0.001$, obtained with Student's t-test. Data based on four independent biological replicates.

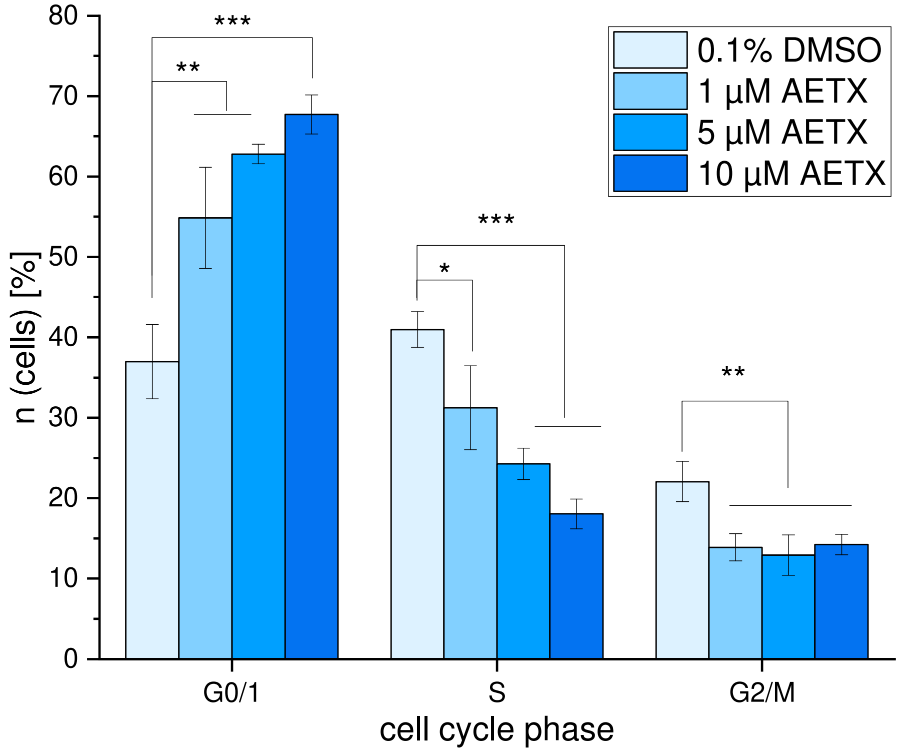

Fig. S7: Distribution of cell cycle phases in HCT116 cell populations treated with 0.1% DMSO, 1, 5, or 10 µM AETX for 24 h. Statistically significant difference to the control indicated with *p<0.05, **p<0.01, ***p<0.001, obtained with Student‘s t-test. Data results from four independent biological replicates.

A

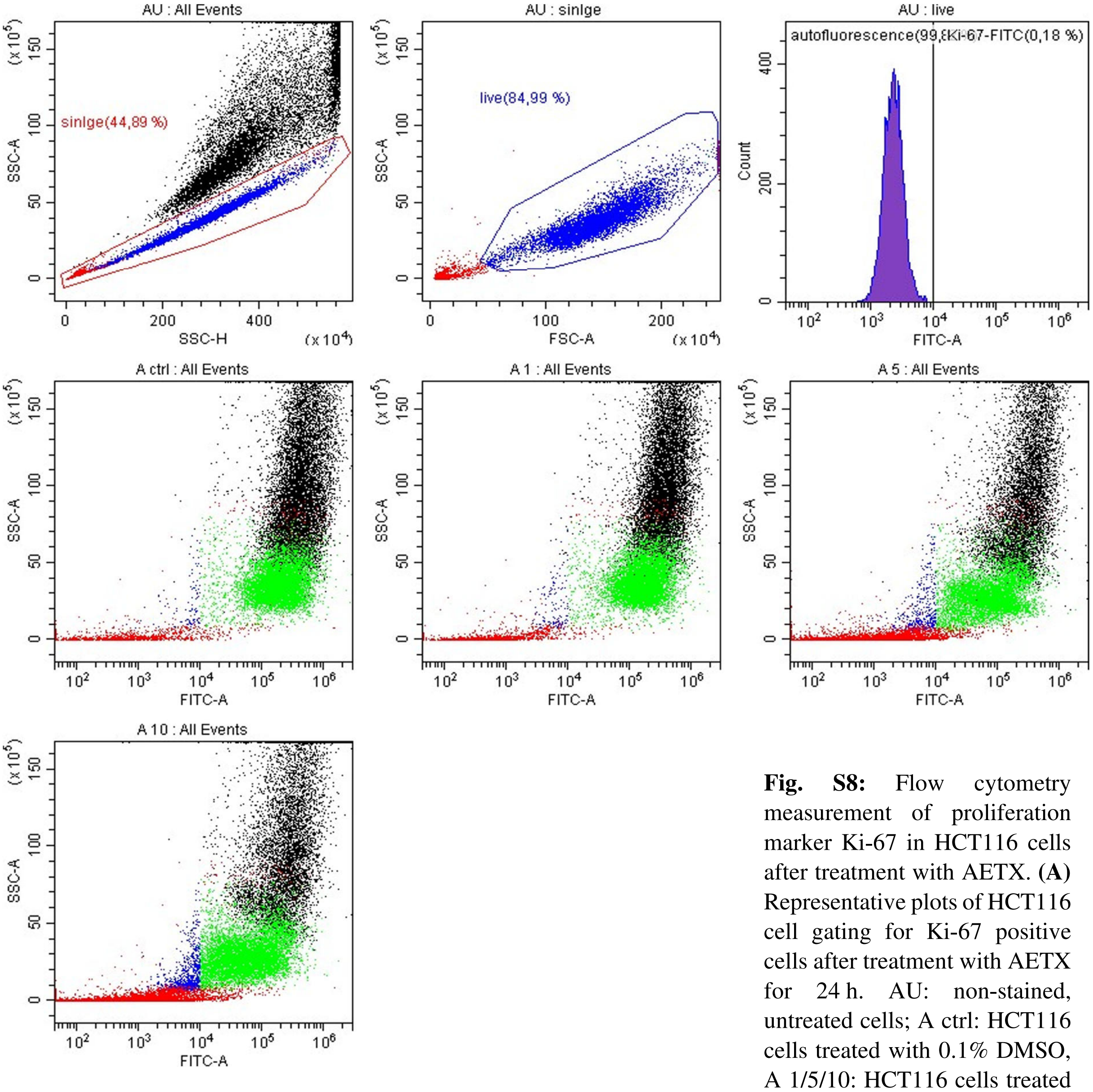

B

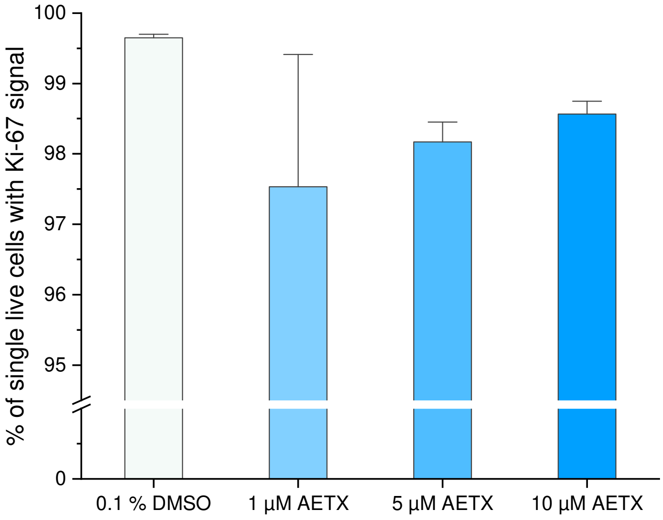

Fig. S8: Flow cytometry measurement of proliferation marker Ki-67 in HCT116 cells after treatment with AETX. **(A)** Representative plots of HCT116 cell gating for Ki-67 positive cells after treatment with AETX for 24 h. AU: non-stained, untreated cells; A ctrl: HCT116 cells treated with 0.1% DMSO, A 1/5/10: HCT116 cells treated with 1, 5, or 10 µM AETX. Gates: red: cell singlets, blue: healthy cells, green: Ki-67-positive cells, black: cells which are excluded from the gates. **(B)** HCT116 cells with Ki-67 signal after 24 h of treatment with 0.1% DMSO, 1, 5, or 10 µM AETX. No statistically significant difference to the control, obtained with Mann-Whitney test. Data obtained from four independent biological replicates. Whiskers indicate standard deviation (1x).

Metabolomics

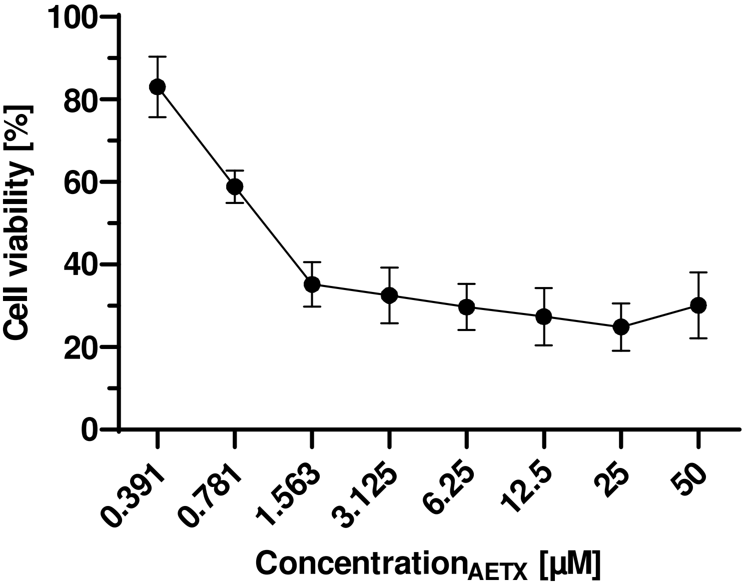

Fig. S9: Mean viability of PC-3 cells treated with several concentrations of AETX as compared to the control. Data determined under use of the MTT assay. Whiskers show standard deviation (1x).

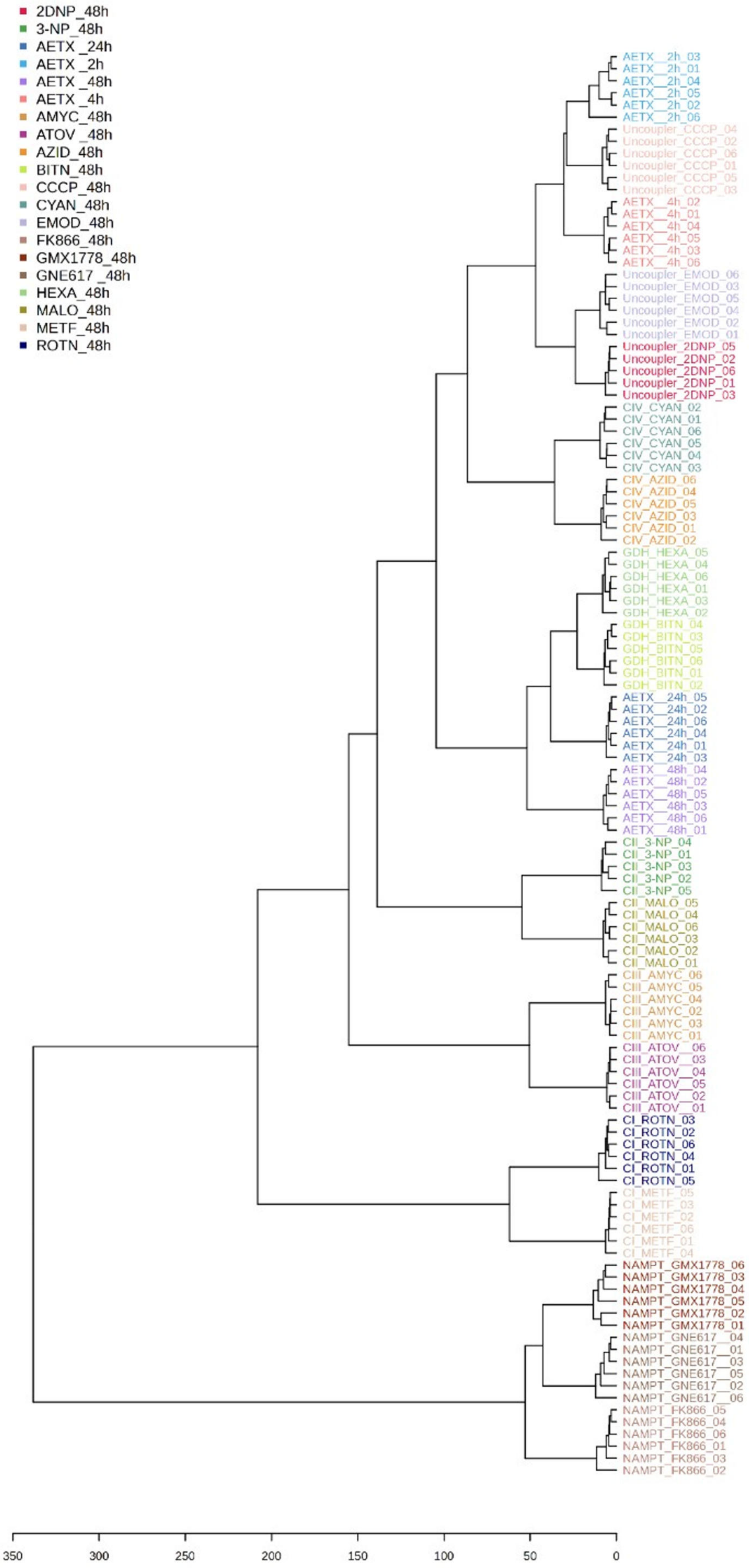

Fig. S10: Hierarchical cluster analysis of metabolic profiles induced by OXPHOS inhibitors, NAMPT inhibitors, GDH inhibi-tors and AETX based on the modulation of the metabolism of prostate cancer cells (PC-3). Data aggregated from six replicates for each experiment.

<u>Compounds:</u>
2DNP - 2,4-dinitrophenol
3-NP - 3-nitropropionic acid
AMYC – antimycin A
ATOV – atovaquone
AZID - sodium azide
BITN – bithionol
CCCP - carbonyl cyanide chlo-rophenylhydrazone
CYAN - potassium cyanide
EMOD – emodin
FK866 - FK866
GMX - GMX1778
GNE - GNE-617
HEXA – hexachlorophene
MALO - malonic acid
METF – metformin
ROTN - rotenone.

<u>MoA:</u>
CPLX I - complex I
CPLX II - complex II
CPLX III - complex III
CPLX IV - complex IV
GDH - glutamate dehydrogenase
NAMPT - nicotinamide phosphor-ribosyltransferase
Uncoupler - uncoupling of oxidative phosphorylation.

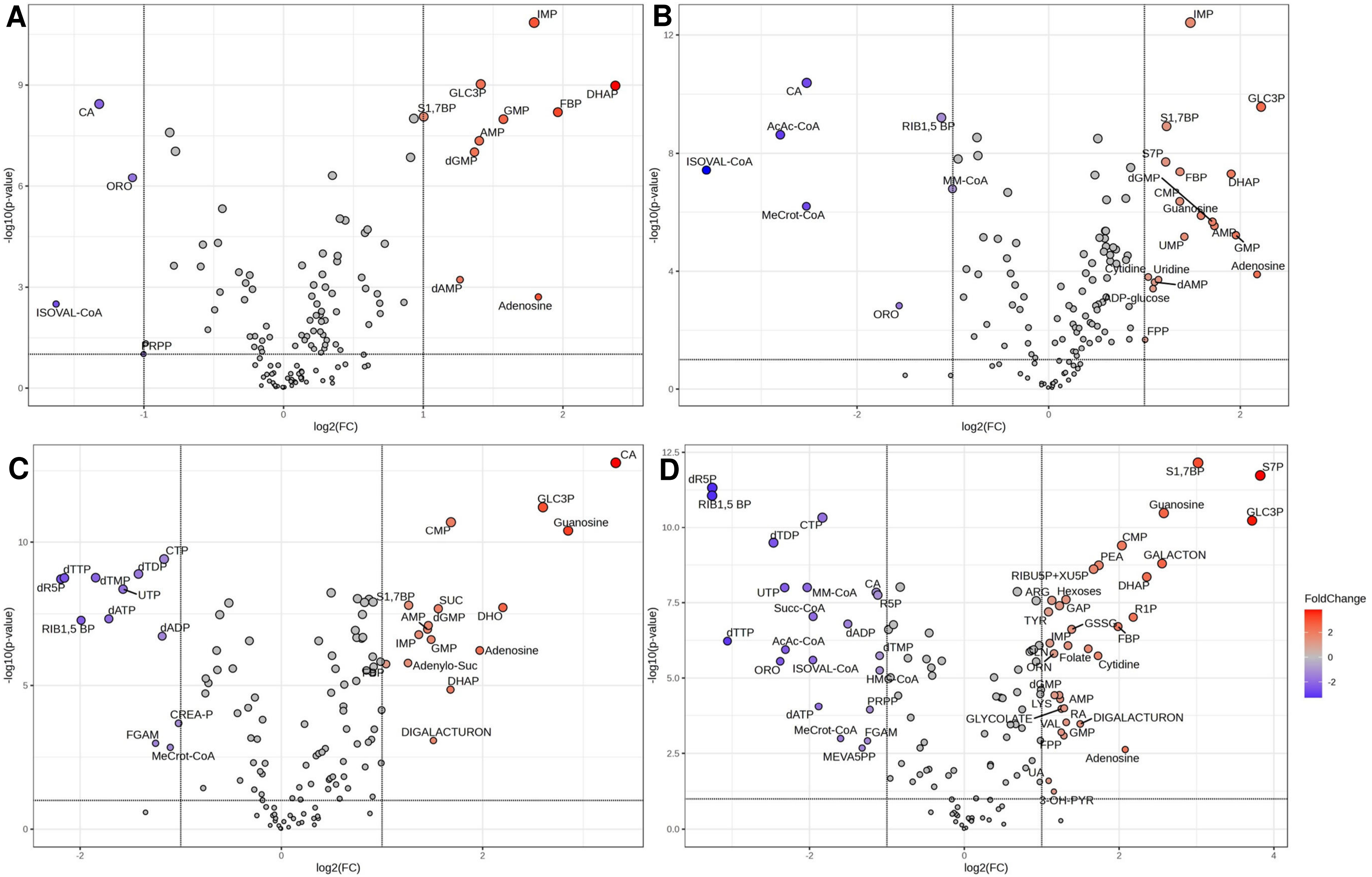

Fig. S11: Log fold changes between metabolites of PC3 cells incubated for 2 h **(A)**, 4 h **(B)**, 24 h **(C)**, and 48 h **(D)** with 0.74 µM AETX or solvent control. Statistical significance indicated in red (higher than control) or blue (lower than control). For abbreviations see supplementary metabolomics data (Excel file).

pKa and logP

Multiset name:	**UV-metric psKa AETX**	Instrument ID:	**T310018**
Analyst:	**RR**		
Quality:	**Good**		
Filename:	**E:\AETX\Experimental\AETX_UV-metric psKa_MeOH - Multiset.t3r**		

Yasuda-Shedlovsky result

Extrapolation type	pKa0%	SD	Intercept	Slope	R²	Ionic strength	Temperature
Yasuda-Shedlovsky	6.87	±0.03	9.70	-85.0455	0.9939	0.163 M	24.9°C

Component assay results

Titration	Methanol weight%	Direction	Result type	Dielectric constant	[H2O]	Ionic strength	Temperature		psKa 1
23I-12006 Points 48 to 91	52.65 %	Up	UV-metric pKa	55.1	23.0 M	0.169 M	25.0°C	☑	6.79
23I-12013 Points 46 to 88	56.46 %	Up	UV-metric pKa	53.3	20.9 M	0.169 M	25.0°C	☑	6.79
23I-13004 Points 4 to 44	58.64 %	Up	UV-metric pKa	52.3	19.8 M	0.157 M	24.9°C	☑	6.77
23I-12006 Points 4 to 46	62.91 %	Up	UV-metric pKa	50.2	17.6 M	0.157 M	24.9°C	☑	6.76

Graphs

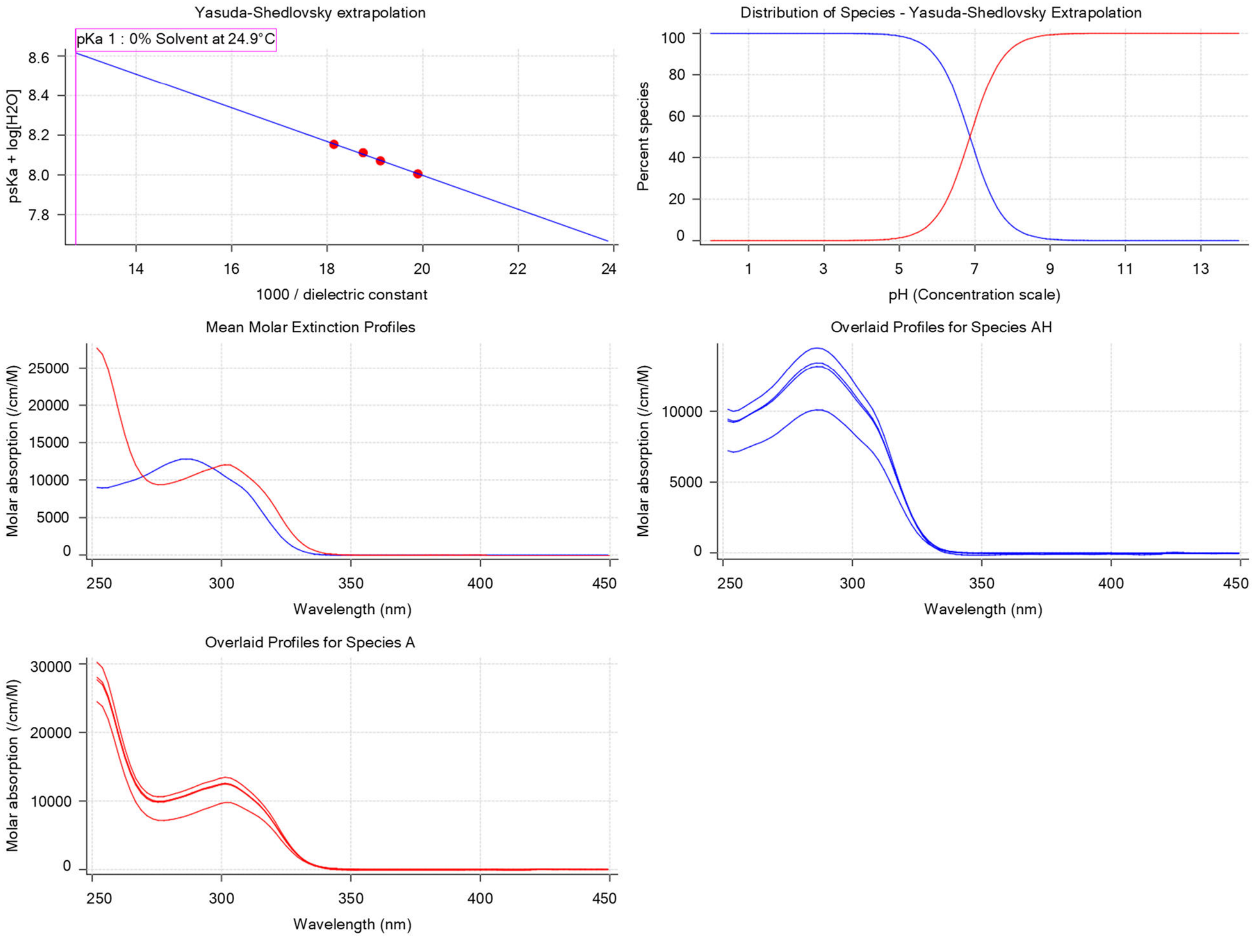

UV-metric psKa 23I-12006 Assay 1 of 3 Quality: Good

Calibration Settings

Setting Value Date/Time changed Imported from
Four-Plus alpha 0.166 12/09/2023 12:34:55 H:\Data 2023\T310018\September\23I-12003_Blank standardisation.t3r
Four-Plus S 0.9994 12/09/2023 12:34:55 H:\Data 2023\T310018\September\23I-12003_Blank standardisation.t3r Four-Plus jH 0.6 12/09/2023 12:34:55 H:\Data 2023\T310018\September\23I-12003_Blank standardisation.t3r

Multiset name: **UV-metric psKa AETX** Instrument ID: **T310018**
Analyst: **RR**
Quality: **Good**
Filename: **E:\AETX\Experimental\AETX_UV-metric psKa_MeOH - Multiset.t3r**

Calibration Settings (continued)

Setting	Value	Date/Time changed	Imported from
Four-Plus jOH	-0.6	12/09/2023 12:34:55	H:\Data 2023\T310018\September\23I-12003_Blank standardisation.t3r
Base concentration factor	1.025	12/09/2023 12:34:55	H:\Data 2023\T310018\September\KHP Multiset 1st September 2023_PASS.t3r
Acid concentration factor	0.988	12/09/2023 12:34:55	H:\Data 2023\T310018\September\23I-12003_Blank standardisation.t3r

UV-metric psKa_Low 23I-12013 Assay 2 of 3 Quality: Good

Calibration Settings

Setting	Value	Date/Time changed	Imported from
Four-Plus alpha	0.166	12/09/2023 17:14:08	H:\Data 2023\T310018\September\23I-12003_Blank standardisation.t3r
Four-Plus S	0.9994	12/09/2023 17:14:08	H:\Data 2023\T310018\September\23I-12003_Blank standardisation.t3r
Four-Plus jH	0.6	12/09/2023 17:14:08	H:\Data 2023\T310018\September\23I-12003_Blank standardisation.t3r
Four-Plus jOH	-0.6	12/09/2023 17:14:08	H:\Data 2023\T310018\September\23I-12003_Blank standardisation.t3r
Base concentration factor	1.025	12/09/2023 17:14:08	H:\Data 2023\T310018\September\KHP Multiset 1st September 2023_PASS.t3r
Acid concentration factor	0.988	12/09/2023 17:14:08	H:\Data 2023\T310018\September\23I-12003_Blank standardisation.t3r

UV-metric psKa_Low 60MeOH 23I-13004 Assay 3 of 3 Quality: Good

Calibration Settings

Setting	Value	Date/Time changed	Imported from
Four-Plus alpha	0.143	13/09/2023 12:48:12	H:\Data 2023\T310018\September\23I-13003_Blank standardisation.t3r
Four-Plus S	0.9997	13/09/2023 12:48:12	H:\Data 2023\T310018\September\23I-13003_Blank standardisation.t3r
Four-Plus jH	0.4	13/09/2023 12:48:12	H:\Data 2023\T310018\September\23I-13003_Blank standardisation.t3r
Four-Plus jOH	-0.4	13/09/2023 12:48:12	H:\Data 2023\T310018\September\23I-13003_Blank standardisation.t3r
Base concentration factor	1.025	13/09/2023 12:48:12	H:\Data 2023\T310018\September\KHP Multiset 1st September 2023_PASS.t3r
Acid concentration factor	0.987	13/09/2023 12:48:12	H:\Data 2023\T310018\September\23I-13003_Blank standardisation.t3r

Reported at: 29/09/2023 14:24:05 Page 2 of 2

Fig. S12: Report of the UV-metric pK_a assessment for AETX.

Sample name:	**AETX**	Experiment start time:	**29/09/2023 18:10:55**
Assay name:	**pH-metric high logP**	Analyst:	**RR**
Assay ID:	**23I-29010**	Instrument ID:	**T311054**
Quality:	**Good**		
Filename:	**E:\AETX\Experimental\23I-29010_AETX_pH-metric high logP.t3r**		

pH-metric Result

logP (neutral XH) 4.74 ±0.07 (n=50)

Sample logD values

pH	AETX logD	Comment
1.000	4.74	
1.200	4.74	Stomach pH
2.000	4.74	
3.000	4.74	
4.000	4.74	
5.000	4.74	
6.000	4.69	
6.500	4.59	
7.000	4.37	
7.400	4.10	Blood pH
8.000	3.58	
9.000	2.61	
10.000	1.61	
11.000	0.61	
12.000	-0.39	

Graphs

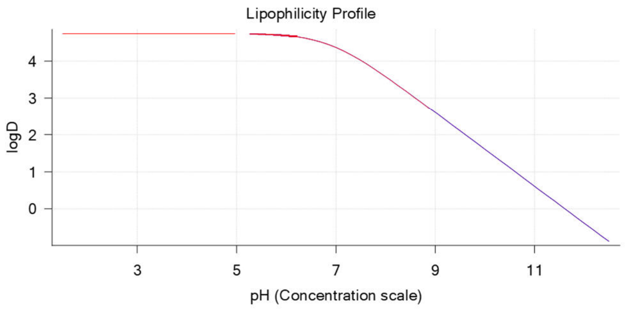

Calibration Settings

Setting	Value	Date/Time changed	Imported from
Four-Plus alpha	0.180	29/09/2023 18:10:55	H:\Data 2023\T311054\September\23I-29004_Blank standardisation.t3r
Four-Plus S	0.9930	29/09/2023 18:10:55	H:\Data 2023\T311054\September\23I-29004_Blank standardisation.t3r
Four-Plus jH	0.8	29/09/2023 18:10:55	H:\Data 2023\T311054\September\23I-29004_Blank standardisation.t3r
Four-Plus jOH	0.0	29/09/2023 18:10:55	H:\Data 2023\T311054\September\23I-29004_Blank standardisation.t3r
Base concentration factor	1.020	29/09/2023 18:10:55	H:\Data 2023\T311054\September\KHP Multiset 1st September 2023_PASS.t3r
Acid concentration factor	0.993	29/09/2023 18:10:55	H:\Data 2023\T311054\September\23I-29004_Blank standardisation.t3r

Reported at: 01/11/2023 13:58:35 Page 1 of 1

Fig. S13: Report of the pH-metric logP assessment for AETX.

Cell biology

Seahorse experiments

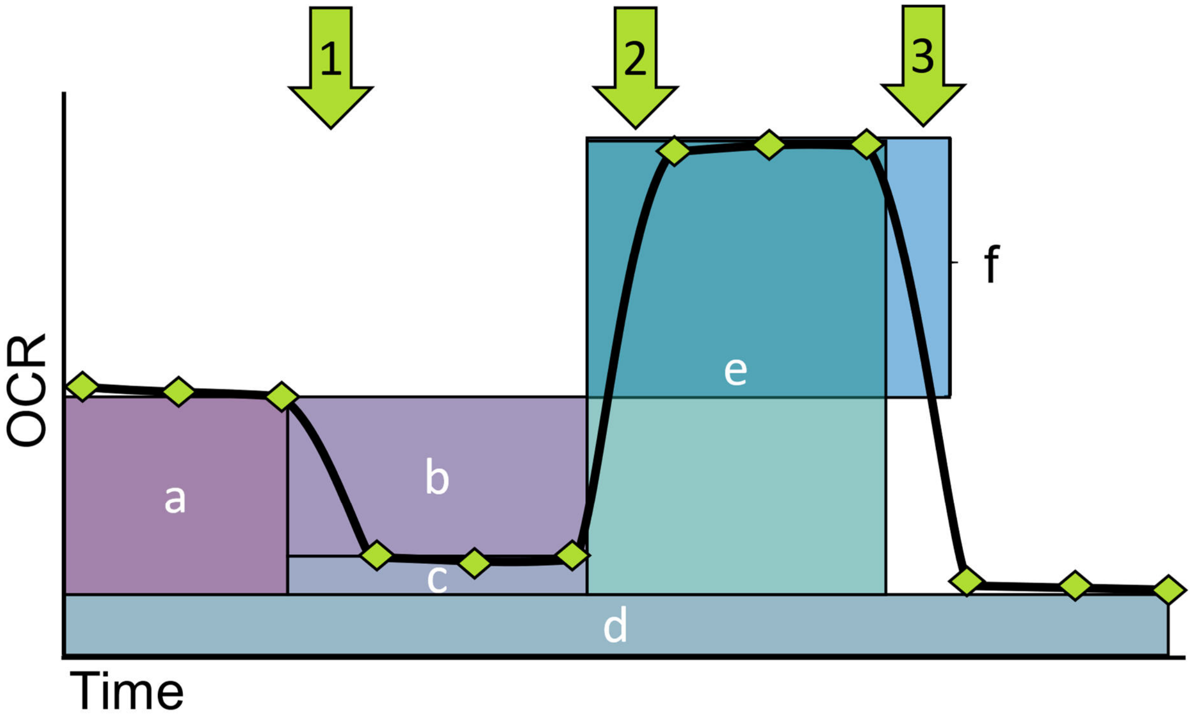

Fig. S14: Sketch of a model oxygen consumption rate assay with the Seahorse FX analyzer. OCR: Oxygen Consumption Rate. Green diamonds indicate single measurements. Green arrows indicate the addition of compounds in the following order: 1: oligomycin to inhibit FoF1-ATPase, 2: FCCP to stimulate respiration, 3: rotenone/antimycin A + Hoechst 33342 to inhibit respiratory chain activity and to stain DNA for subsequent normalization on cell count. Areas indicate endpoints accessible through this approach: a: basal respiration, b: mitochondrial ATP production, c: proton leak, d: non-mitochondrial oxygen consumption, e: maximal respiration, f: spare respiratory capacity.

PER

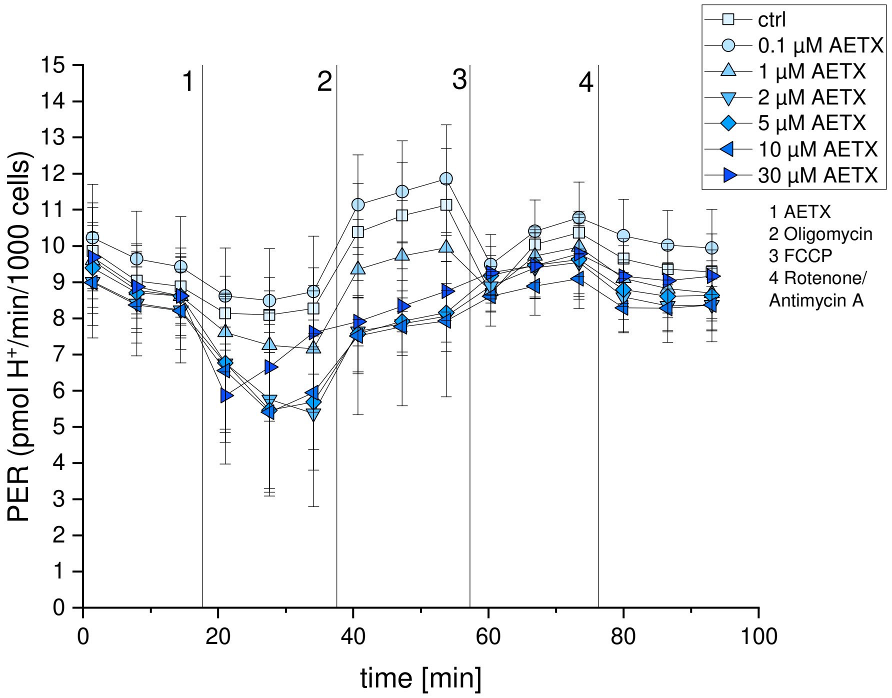

Fig. S15: Proton Efflux Rate (PER) measurement of fibroblasts after acute stimulation with AETX. □: solvent control (0.1% DMSO), ○: 0.1 µM AETX, △: 1 µM AETX, ▽: 2 µM AETX, ◇: 5 µM AETX, ◁: 10 µM AETX, ▷: 30 µM AETX. Addition of 1: AETX, 2: oligomycin, 3: FCCP, 4: rotenone/antimycin A + Hoechst 33342. Whiskers indicate standard deviation (1x).

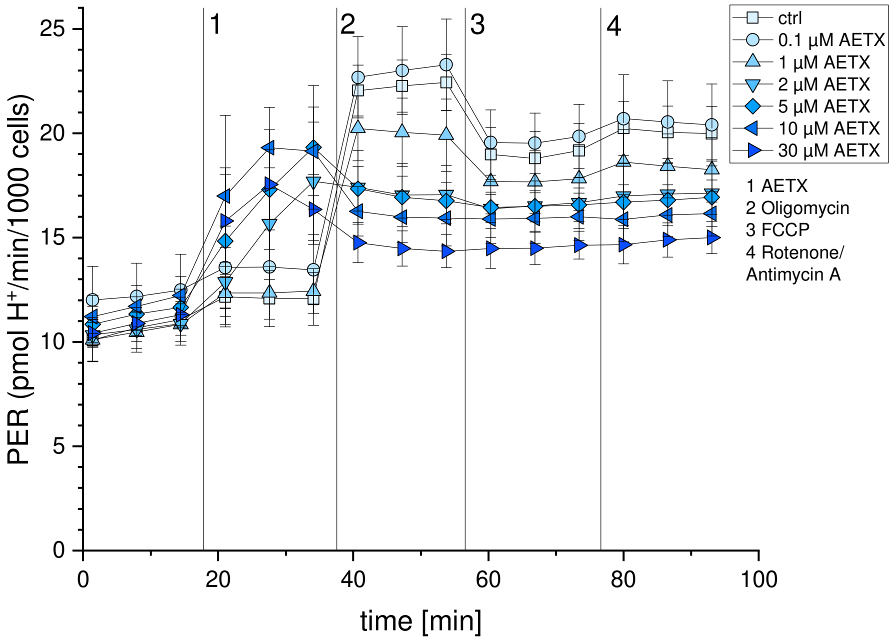

Fig. S16: Proton Efflux Rate (PER) measurement of HeLa cells after acute stimulation with AETX. □: solvent control (0.1% DMSO), ○: 0.1 µM AETX, △: 1 µM AETX, ▽: 2 µM AETX, ◇: 5 µM AETX, ◁: 10 µM AETX, ▷: 30 µM AETX. Addition of 1: AETX, 2: oligomycin, 3: FCCP, 4: rotenone/antimycin A + Hoechst 33342. Whiskers indicate standard deviation (1x).

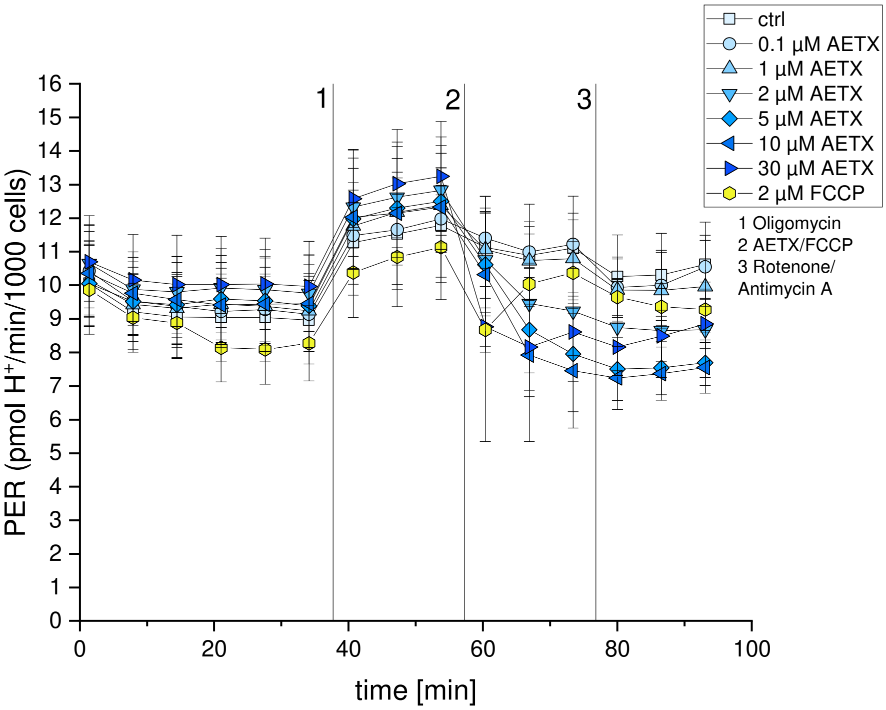

Fig. S17: Proton Efflux Rate (PER) measurement of fibroblasts after acute stimulation with AETX or FCCP subsequent to FoF1-ATPase inhibition. □: solvent control (0.1% DMSO), ○: 0.1 µM AETX, △: 1 µM AETX, ▽: 2 µM AETX, ◇: 5 µM AETX, ◁: 10 µM AETX, ▷: 30 µM AETX, ⬡: 2 µM FCCP. Addition of 1: oligomycin, 2: AETX or 2 µM FCCP, 3: rotenone/antimycin A + Hoechst 33342. Whiskers indicate standard deviation (1x).

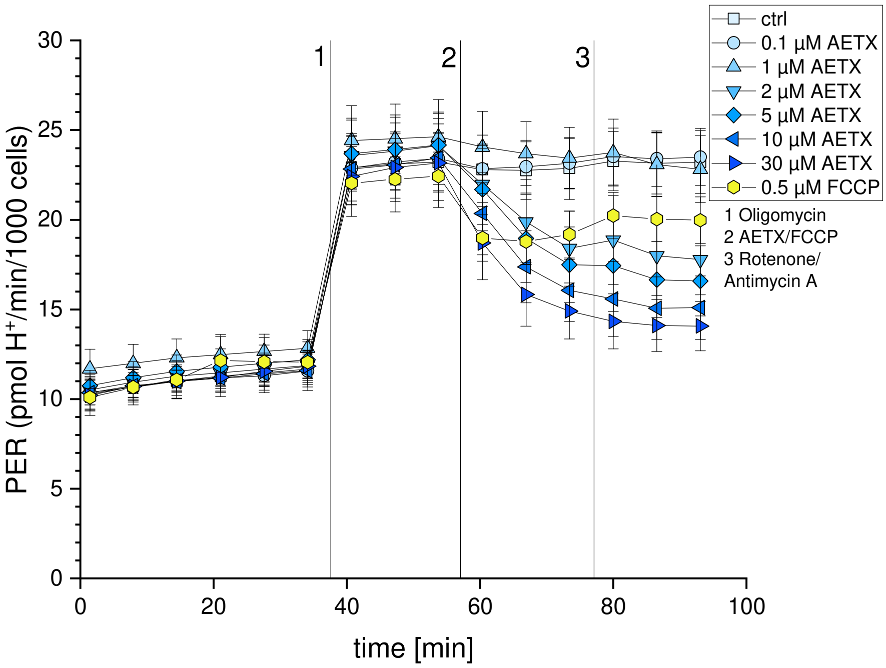

Fig. S18: Proton Efflux Rate (PER) measurement of HeLa cells after acute stimulation with AETX or FCCP subsequent to FoF1-ATPase inhibition. □: solvent control (0.1% DMSO), ○: 0.1 µM AETX, △: 1 µM AETX, ▽: 2 µM AETX, ◇: 5 µM AETX, ◁: 10 µM AETX, ▷: 30 µM AETX, ⬡: 0.5 µM FCCP. Addition of 1: oligomycin, 2: AETX or 0.5 µM FCCP, 3: rotenone/antimycin A + Hoechst 33342. Whiskers indicate standard deviation (1x).

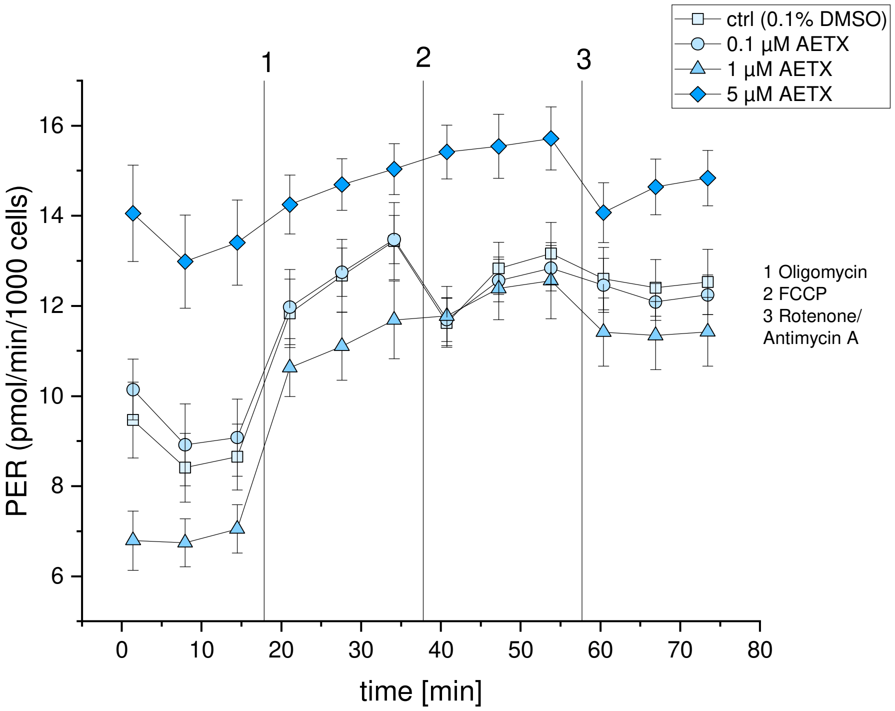

Fig. S19: Proton Efflux Rate (PER) measurement of fibroblasts after 24 h of treatment with AETX. □: solvent control (0.1% DMSO), ○: 0.1 µM AETX, △: 1 µM AETX, ◇: 5 µM AETX. Addition of 1: oligomycin, 2: FCCP, 3: rotenone/antimycin A + Hoechst 33342. Whiskers indicate standard deviation (1x).

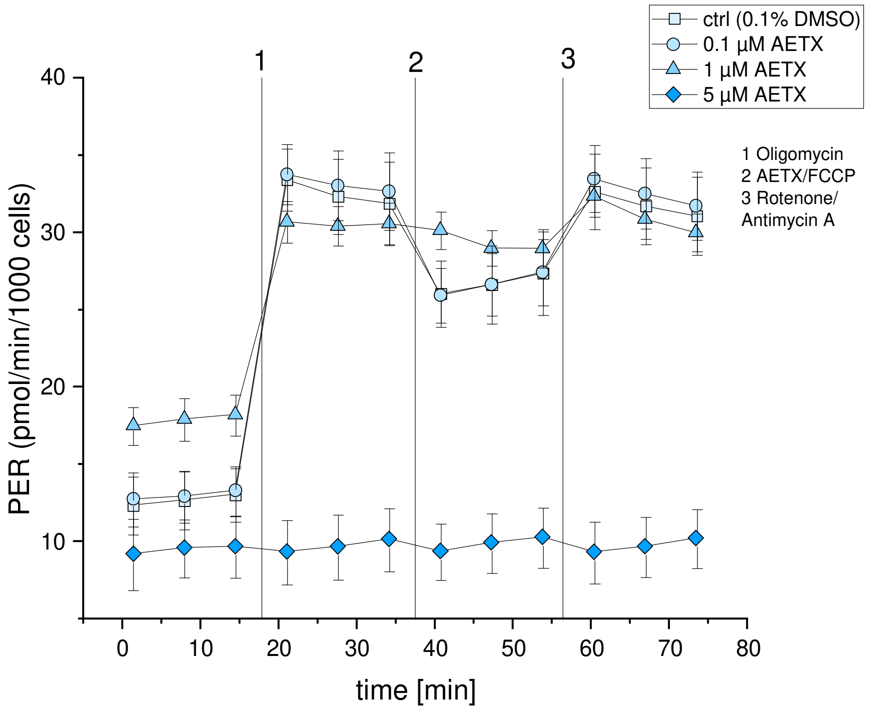

Fig. S20: Proton Efflux Rate (PER) measurement of HeLa cells after 24 h of treatment with AETX. □: solvent control (0.1% DMSO), ○: 0.1 µM AETX, △: 1 µM AETX, ◇: 5 µM AETX. Addition of 1: oligomycin, 2: FCCP, 3: rotenone/antimycin A + Hoechst 33342. Whiskers indicate standard deviation (1x).

Proton leak and coupling efficiency

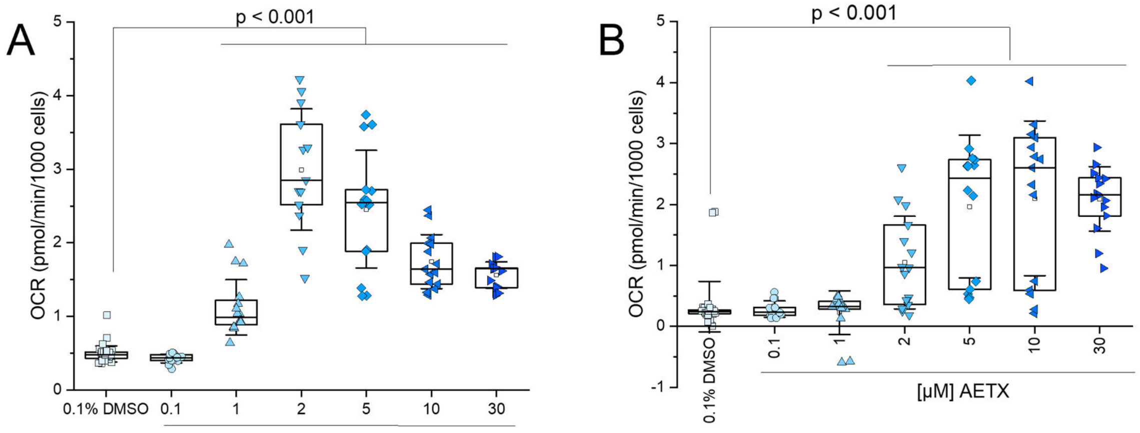

Fig. S21: Proton leak in **(A)** HeLa cells, and **(B)** fibroblasts, both treated with AETX. □: solvent control (0.1% DMSO), ○: 0.1 µM AETX, △: 1 µM AETX, ▽: 2 µM AETX, ◇: 5 µM AETX, ◁: 10 µM AETX, ▷: 30 µM AETX. Statistically significant difference to control obtained with Student‘s t-test. Whiskers indicate standard deviation (1x). Boxes indicate 25-75 percentile with the median as line and mean as open square.

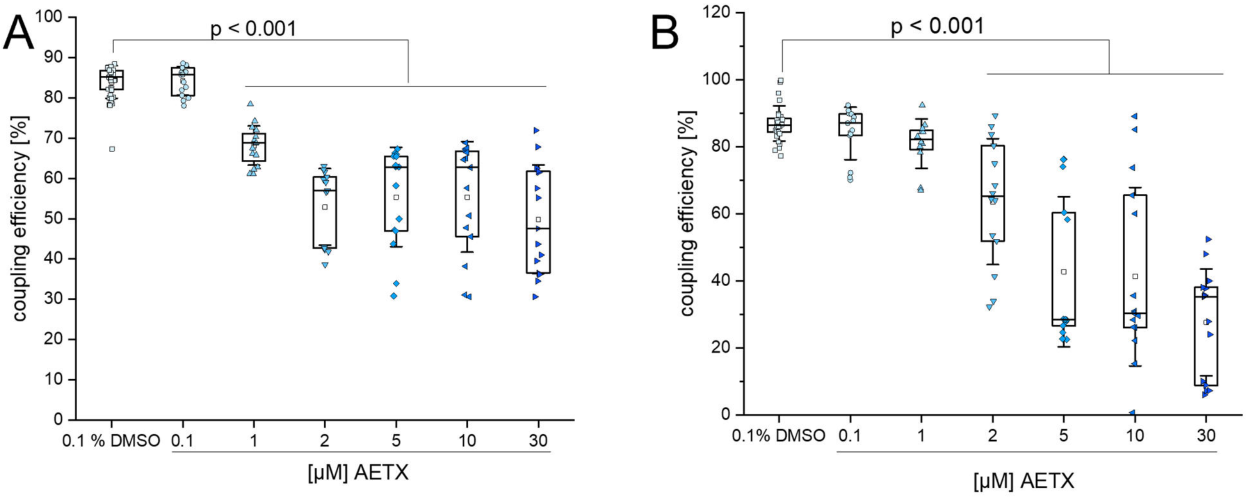

Fig. S22: Coupling efficiency in **(A)** HeLa cells, and **(B)** fibroblasts, both treated with AETX. □: solvent control (0.1% DMSO), ○: 0.1 µM AETX, △: 1 µM AETX, ▽: 2 µM AETX, ◇: 5 µM AETX, ◁: 10 µM AETX, ▷: 30 µM AETX. Statistically significant difference to control obtained with Student‘s t-test. Whiskers indicate standard deviation (1x). Boxes indicate 25-75 percentile with the median as line and mean as open square.

Supporting OCR I

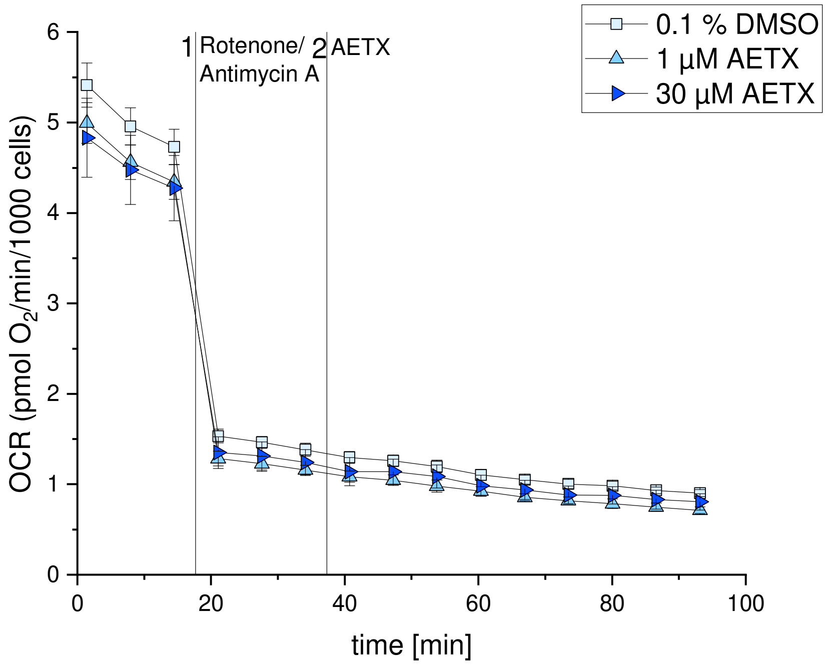

Fig. S23: Oxygen consumption rate (OCR) in HeLa cells after inhibition of the respiratory chain with rotenone/ Antimycin A (1). After the inhibition, AETX was added (2). □: solvent control (0.1% DMSO), △: 1 µM AETX, ▷: 30 µM AETX. Cave: biological replicate = 1.

Cell biology – total ATP level

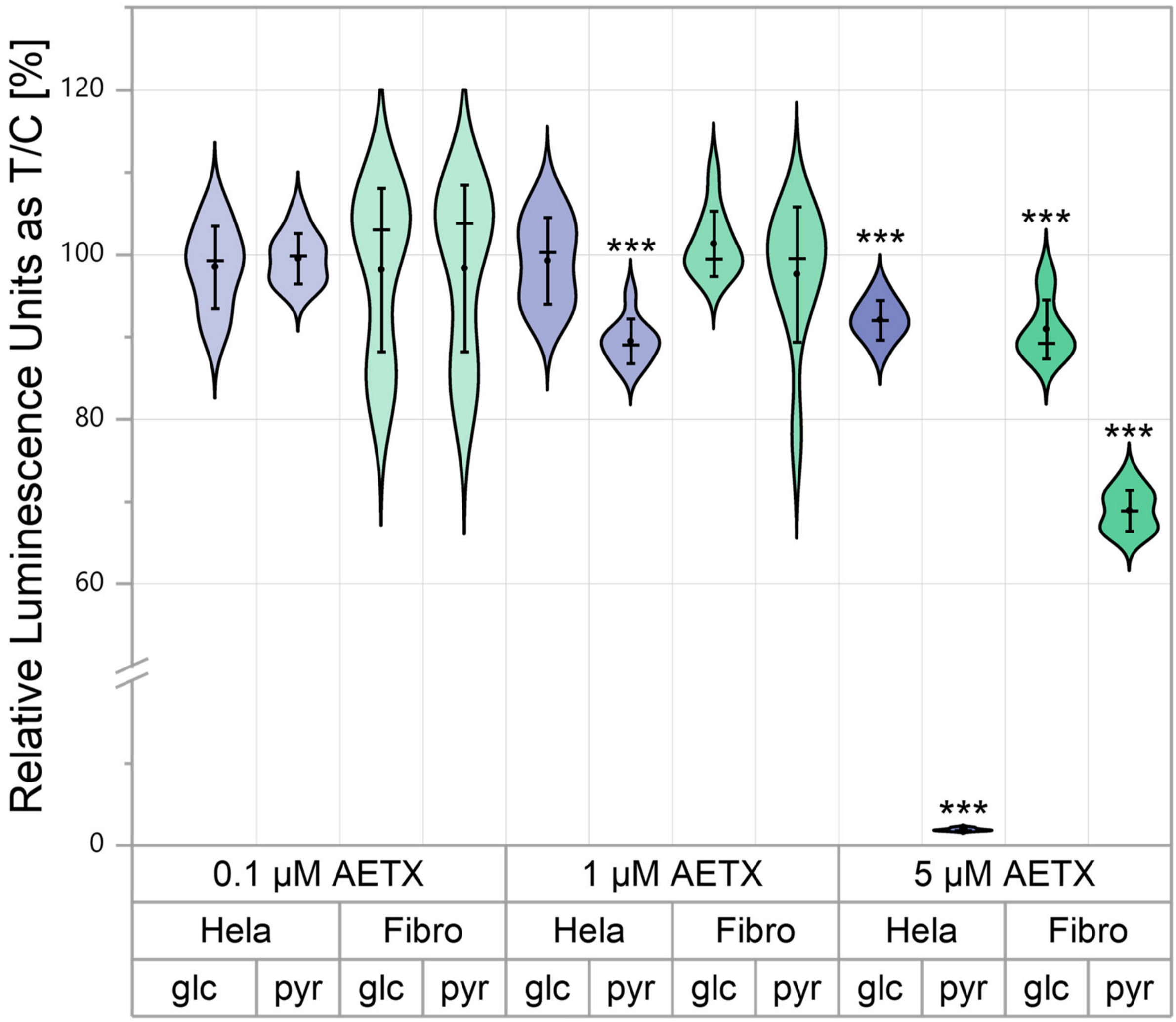

Fig. S24: Mean relative luminescence units of AETX-treated HeLa cells (Hela, violet) and fibroblasts (Fibro, green) in dependence of different media supply. Data is normalized to the control. glc: glucose containing medium. pyr: pyruvate containing medium. Statistically significant difference to the control indicated with *** $p < 0.001$, obtained with Student's t-test (HeLa cells) or Mann-Whitney test (fibroblasts). Mean indicated as black dot and the median as line. Standard deviation (1x) indicated as whiskers. Data obtained from three independent biological replicates.

Seahorse – supporting OCR II

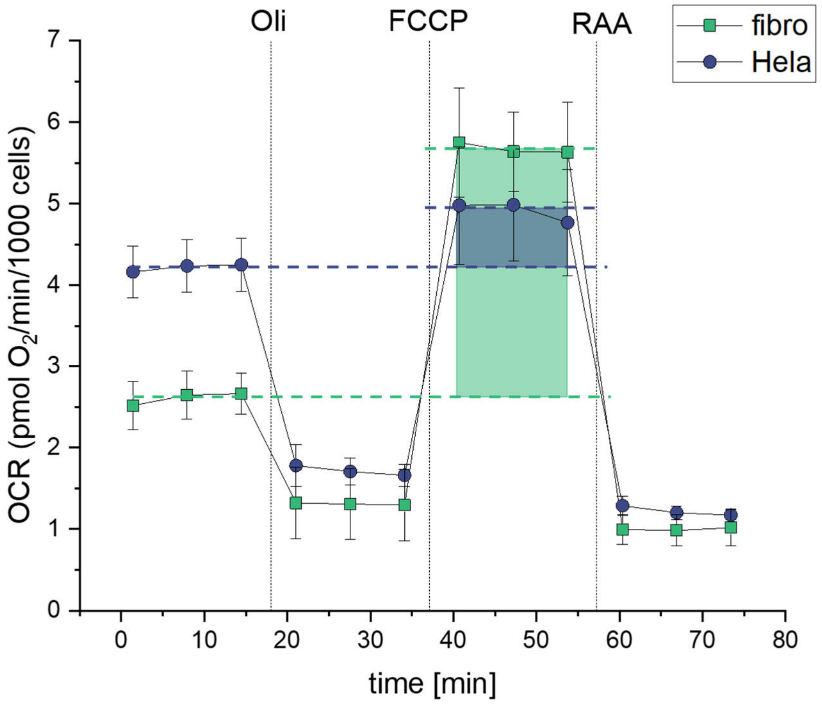

Fig. S25: Mean oxygen consumption rate (OCR) of fibroblasts (fibro, green □) and HeLa cells (Hela, blue ○). Cells were treated with 0.1% DMSO and subsequently with oligomycin (Oli), FCCP and rotenone/antimycin A + Hoechst 33342 (RAA). Whiskers indicate standard deviation (1x). Spare respiratory capacity indicated as colored areas, green: fibroblasts, blue: HeLa cells.

Cell biology – enhanced cytotoxicity

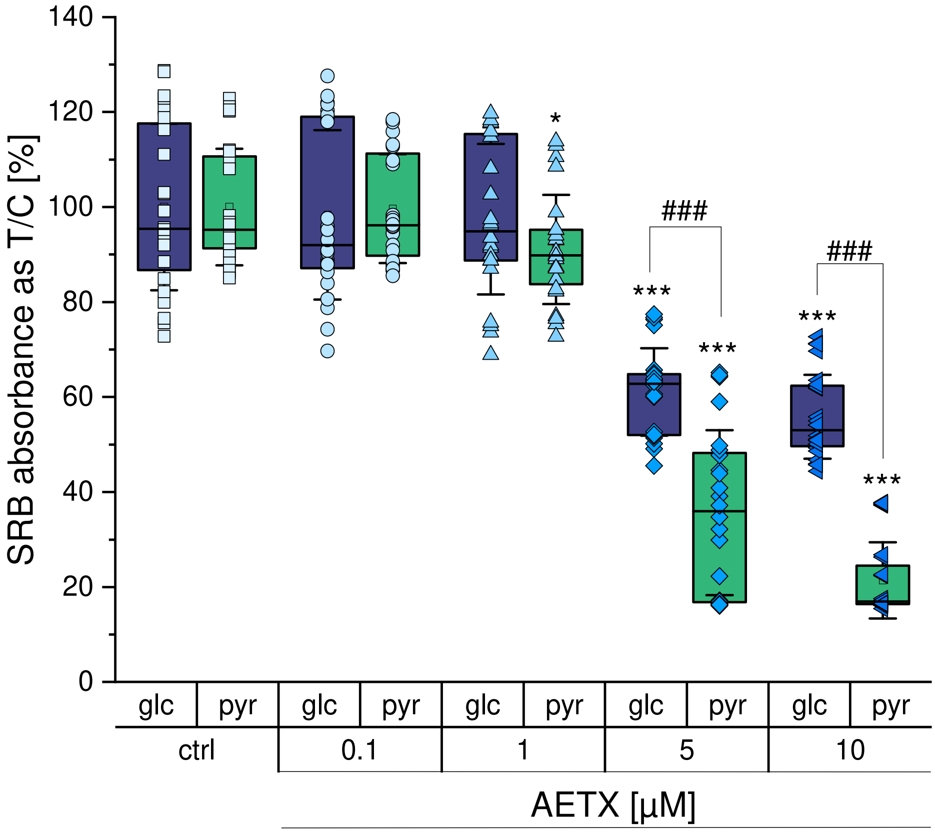

Fig. S26: Mean SRB absorbance of AETX-treated HCT116 cells in dependence of different media supply. Data is normalized to the control. Green: pyruvate containing medium (pyr), violet: glucose containing medium (glc). □: 0.1% DMSO (solvent control), ○: 0.1 µM, △: 1 µM, ◇: 5 µM, ◁: 10 µM. Boxes indicate 25-75 percentile with the median as line. Standard deviation (1x) indicated as whiskers. ### p < 0.001 comparison between the different media, */ *** p < 0.05/0.001 comparison to the respective solvent control. Statistical results obtained with Mann-Whitney test. Data from six independent biological replicates.

Cell biology – ROS

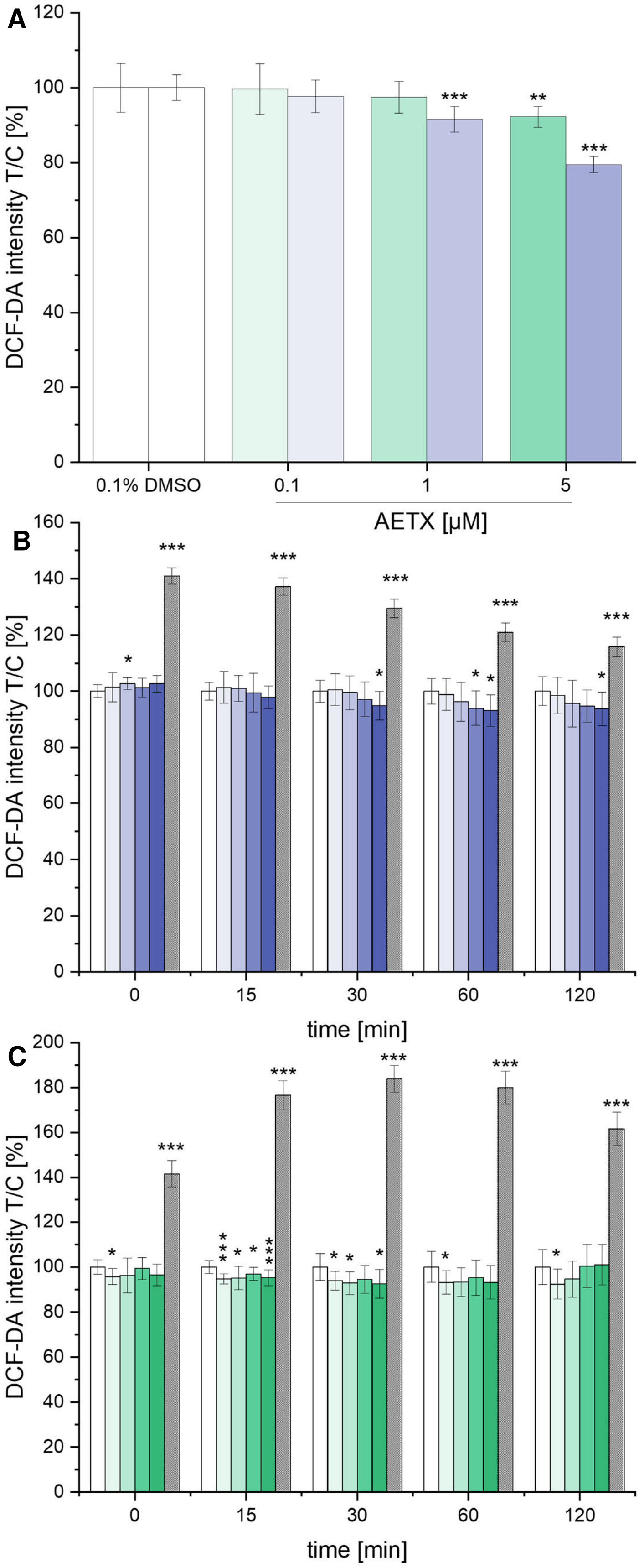

Fig. S27: Effect of AETX on ROS formation detected with DCF-DA. **(A)** Mean DCF-DA intensity of AETX-treated Hela cells and fibroblasts after 24 h incubation normalized to control. **(B)** Kinetic of mean DCF-DA intensity of AETX-treated HeLa cells, and **(C)** fibroblasts normalized to the control. Grey bars: positive control (H_2O_2). Panel **A – C**: violet: HeLa cells, green: fibroblasts. Increasing shades indicate increasing concentrations of AETX (0.1% DMSO (control), 0.1, 1, 5, and 10 µM AETX); Statistically significant difference to the control indicated with */**/*** $p < 0.05/ 0.01/ 0.001$, obtained with Student's t-test. Whiskers indicate standard deviation (1x).

Biophysics – artificial lipid bilayer conductance

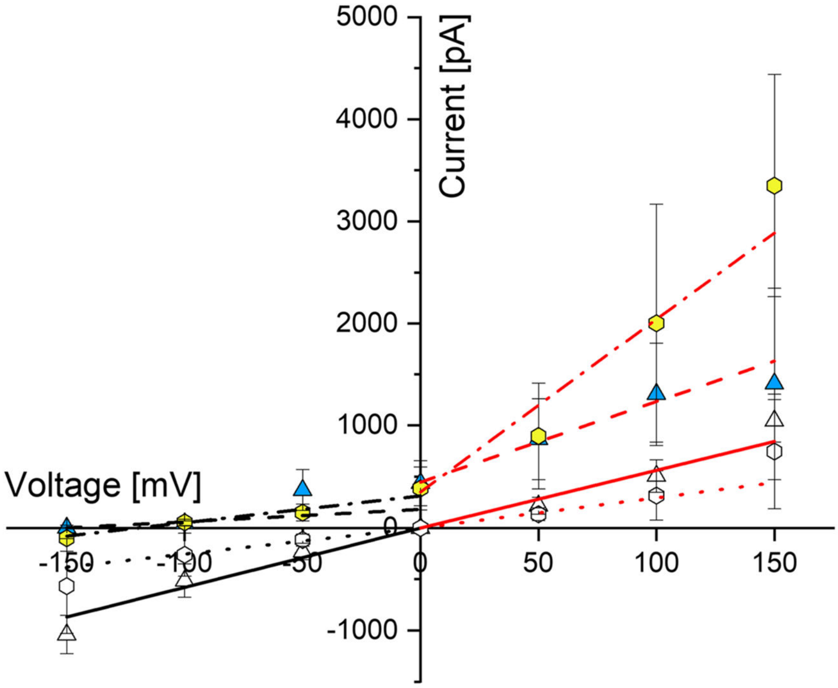

Table 1: linear fit from negative voltage data

Equation	y = a + b*x			
Data	1 µM AETX + pH 7	1 µM AETX + pH 3	1 µM FCCP + pH 7	1 µM FCCP + pH 3
y-intersept	0,00709 ± 0,88065	181,18104 ± 91,73163	0,00188 ± 0,30809	312,96955 ± 44,98594
slope (= conductance [nS])	5,79826 ± 0,74218	1,2041 ± 0,62347	2,54865 ± 0,30425	2,61474 ± 0,45889
sum of squared errors	1,55117	3,43574	0,18984	0,30015
Pearson R	0,98401	0,80682	0,98605	0,97055
R-square	0,96827	0,65096	0,97229	0,94197
cor. R-square	0,95241	0,47643	0,95843	0,91296

Table 2: linear fit from positive voltage data

Equation	y = a + b*x			
Data	1 µM AETX + pH 7	1 µM AETX + pH 3	1 µM FCCP + pH 7	1 µM FCCP + pH 3
y-intersept	-0,0072 ± 0,9246	447,12931 ± 42,85274	-0,00233 ± 0,42365	351,65624 ± 106,87428
slope (=conductance [nS])	5,62504 ± 0,81496	7,87048 ± 0,85199	2,94302 ± 0,44799	16,89456 ± 2,99134
sum of squared errors	1,70983	0,08256	0,35896	0,54362
Pearson R	0,97965	0,98848	0,9776	0,97005
R-square	0,95971	0,9771	0,95571	0,941
cor. R-square	0,93957	0,96565	0,93357	0,9115

Fig. S28: Current-voltage plot displaying translocation of protons across a protein-free artificial planar lipid bilayer. △: 1 µM AETX, ⬡: 1 µM FCCP. Yellow/blue: measurement in water with pH 3 on the amplifier side. White: control measurement in HEPES-buffered solution at pH 7. Whiskers indicate standard deviation (1x). Black lines: linear fits obtained from negative voltage data. Red lines: linear fits obtained from positive voltage data. Respective numerical values (i.e., respective conductance values) displayed in tables 1 and 2 underneath.

Chemistry – purity of AETX, dn-AETX, m-AETX

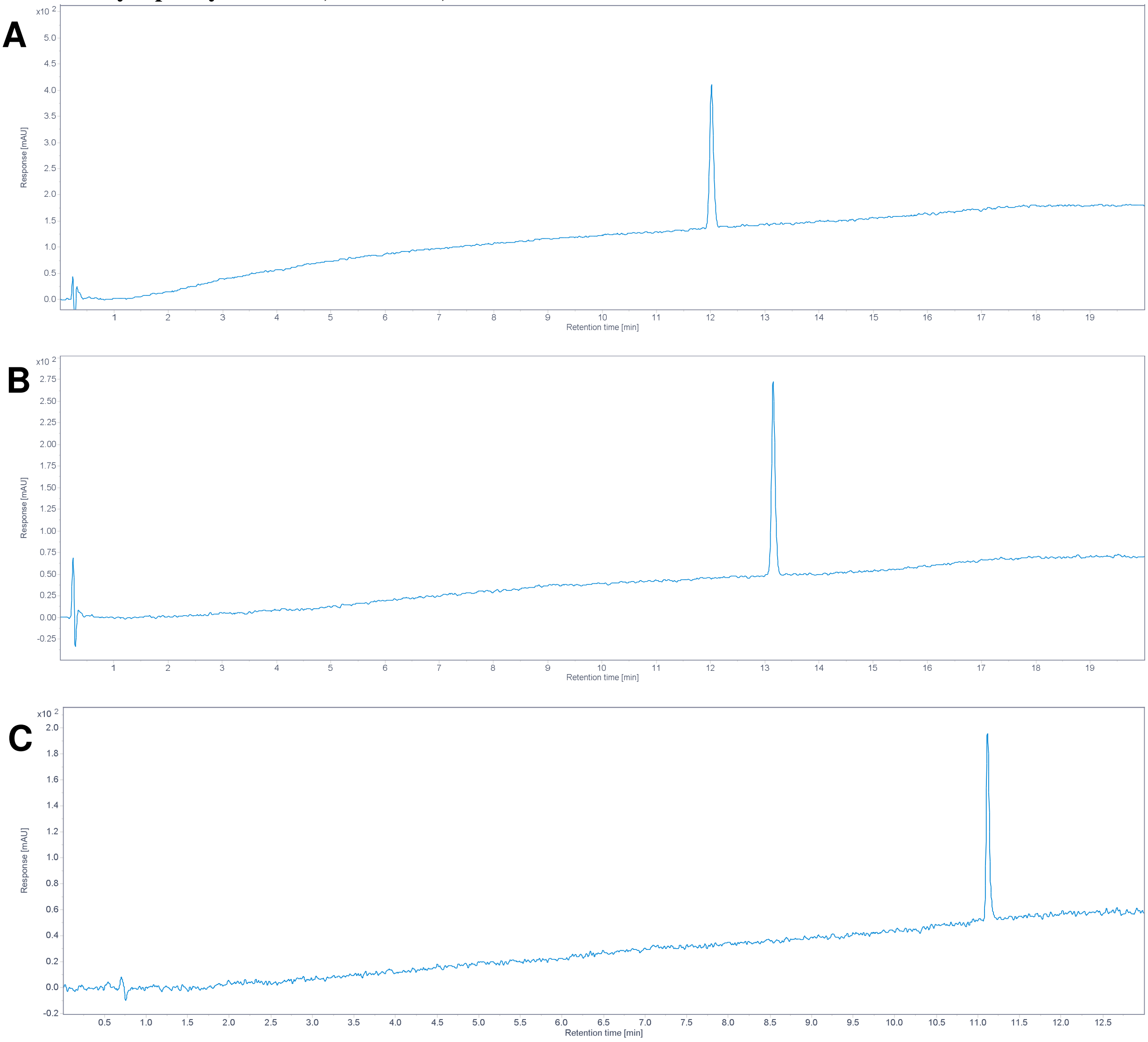

Fig. S29: HPLC-UV chromatogram of AETX (**A**), dn-AETX (**B**) and m-AETX (**C**) at 210 nm. Purity of all compounds >99.5%.

Chemistry – structure confirmation of m-AETX

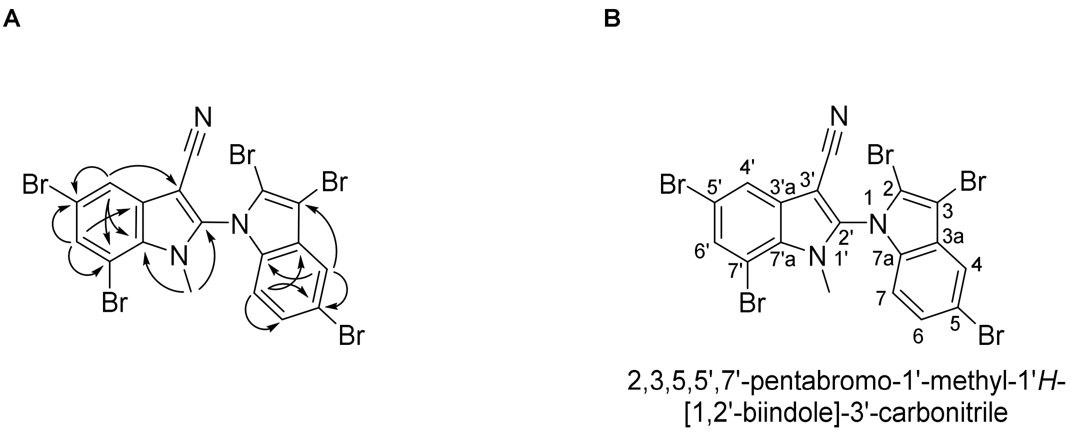

Fig. S30: Structure confirmation with (**A**) key HMBC correlations, (**B**) numbering and IUPAC name.

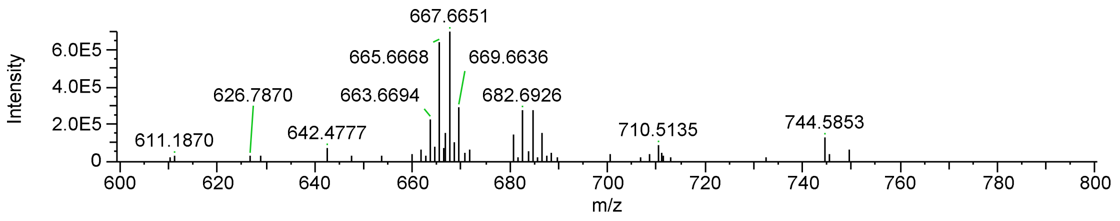

Fig. S31: HRMS spectrum of m-AETX (pos. mode). $[M+H]^+$ at *m/z* 667.6651, $C_{18}H_9N_3{}^{79}Br_2{}^{81}Br_3$ (calc.667.6652, Δ 0.1 ppm)

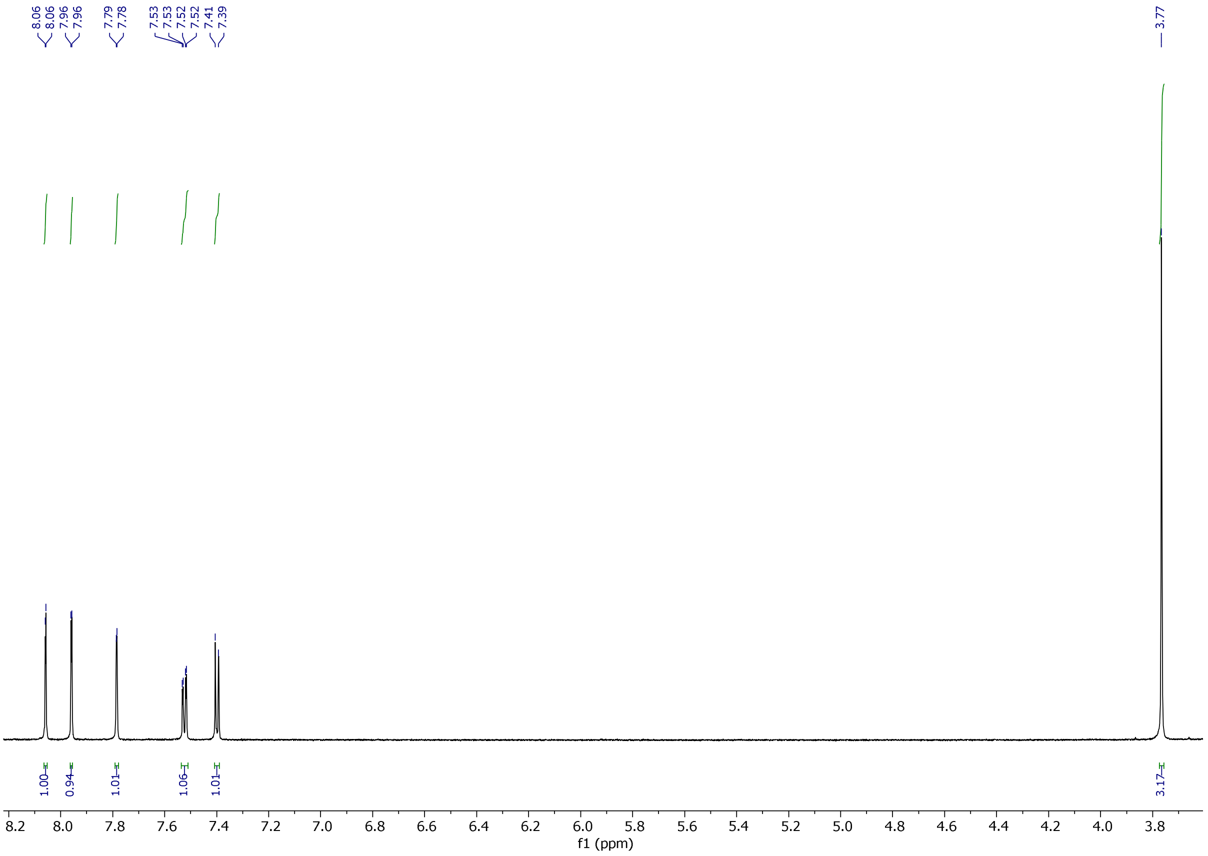

Fig. S32: ^{1}H NMR spectrum (700 MHz) of m-AETX in DMSO-d_6 (3.5 to 8.2 ppm).

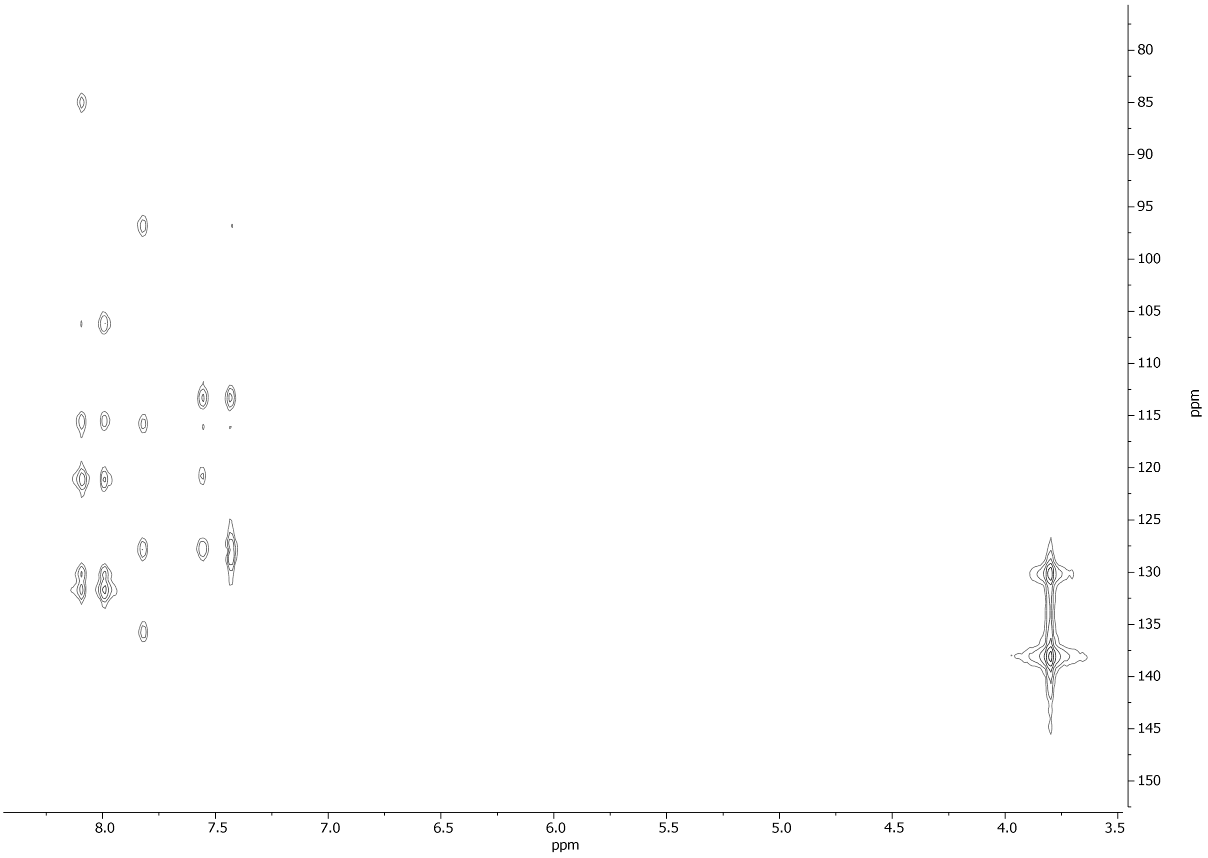

Fig. S33: ^{13}C-HMBC NMR spectrum (700 MHz) of m-AETX in DMSO-d_6 (3.4 to 8.4 ppm).

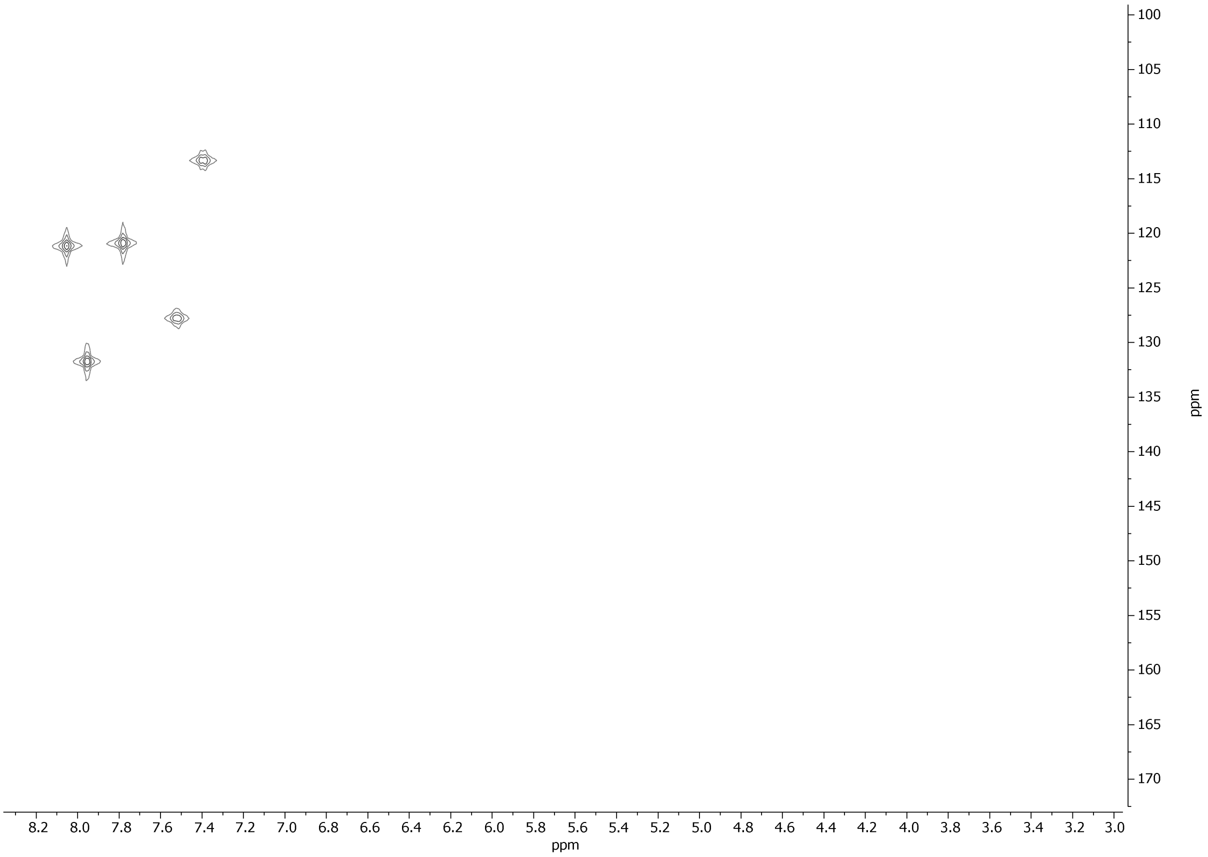

Fig. S34: ^{13}C-HSQC NMR spectrum (700 MHz) of m-AETX in DMSO-d_6 (3.0 to 8.2 ppm).
